# A Unique m6A-Dependent Restriction Endonuclease from an Archaeal Virus

**DOI:** 10.1128/spectrum.04262-22

**Published:** 2023-03-22

**Authors:** Xueling Lu, Fengtao Huang, Rui Cheng, Bin Zhu

**Affiliations:** a Key Laboratory of Molecular Biophysics, the Ministry of Education, College of Life Science and Technology, Huazhong University of Science and Technology, Wuhan, Hubei, China; b Shenzhen Huazhong University of Science and Technology Research Institute, Shenzhen, China; Institute of Microbiology Chinese Academy of Sciences

**Keywords:** N6-methyladenine, archaeal virus, epigenetics, m6A-dependent endonuclease, restriction enzyme

## Abstract

Prokaryotes possess numerous diverse defense systems to resist viral infections, while some viruses have also evolved antiviral defense systems to exclude other viruses in cases of multiple infections. Here, we report the first virus-derived modification-dependent restriction endonuclease (HHPV4I) from the archaeal virus HHPV4 (Haloarcula hispanica pleomorphic virus 4). HHPV4I contains an SRA domain, a winged helix (wH) domain, and an HNH domain; recognizes the Gm6ATC site; and specifically binds to Gm6ATC site-containing DNA. Both the wH domain and the HNH domain are responsible for DNA binding. Unlike the well-known m6A-specific restriction enzyme DpnI, HHPV4I only efficiently cleaves DNA with a fully methylated Gm6ATC site and cleaves DNA both upstream and downstream of the Gm6ATC sites on both DNA strands. Furthermore, HHPV4I preferentially cleaves DNA between VR bases (V = A/G/C, R = A/G) 4 to 20 nt away from the Gm6ATC site. Thus, the cleavage pattern of HHPV4I is distinct from those of all of the presently characterized restriction endonucleases. Mutations in the wH domain of HHPV4I do not alter m6A-dependent endonuclease activity, but they decrease recognition sequence specificity, thus expanding the cleaving capacity to more m6A-containing DNA sequences. The wH domain provides a target for searching, developing, and engineering novel m6A-dependent endonucleases.

**IMPORTANCE** Many modification-dependent restriction endonucleases (MDREs) were identified in prokaryotes and recognized modified cytosine bases, such as 5-methylcytosine (5mC), 5-hydroxymethylcytosine (5hmC), and glucosyl-5-hydroxymethylcytosine (g5hmC). The first virus-derived MDRE (HHPV4I) from the archaeal virus HHPV4 was identified in this study. The viral MDRE suggested a new strategy employed by the virus to exclude other viruses in the case of multiple replications. HHPV4I is a novel N6-methyladenine (m6A)-dependent restriction endonuclease, while the cleavage pattern of HHPV4I is distinct from the well-known m6A-dependent restriction endonuclease DpnI. HHPV4I recognizes Gm6ATC sites and cleaves DNA both upstream and downstream of the Gm6ATC sites on both DNA strands. It preferentially cleaves DNA between VR bases (V = A/G/C, R = A/G) 4 to 20 nt away from the Gm6ATC sites. Furthermore, mutations in the HHPV4I wH domain can alter the sequence specificity without impeding the m6A-dependent DNA cleavage activity, providing a target for engineering more m6A-dependent endonucleases with different sequence specificities.

## INTRODUCTION

Modification-dependent restriction endonucleases (MDREs) are commonly present in bacteria, and they mediate antiviral immunity by targeting bacteriophage genomes containing modified bases such as 5-methylcytosine (5mC), 5-hydroxymethylcytosine (5hmC), glucosyl-5-hydroxymethylcytosine (g5hmC), and N6-methyladenine (m6A). To date, many MDREs have been characterized. These enzymes contain different DNA binding domains and catalytic domains; they may recognize different modified bases and show different cleavage properties (1 to 3). Among these characterized MDREs, most recognize DNA containing modified cytosine bases such as the 5mC modification (1). SRA domains, which were first characterized as 5mC-dependent DNA binding domains in mammals, are also ubiquitously present in bacteria and are usually fused to endonuclease domains functioning as cytosine modification-dependent restriction endonucleases (2, 4 to 6). In recent years, much attention has been paid to SRA domain-containing MDREs (2). Various SRA domain-containing endonucleases have been characterized, such as SRA-PD-(D/E)XK endonucleases MspJI and its homologs (7 to 9), SRA-HNH endonuclease TagI (10), and PD-(D/E)XK-SRA endonuclease PvuRts1I and its homologs (11). MspJI and TagI recognize the 5mC and 5hmC modification DNA, but not the g5hmC modification DNA, whereas PvuRts1I recognizes the 5hmC and g5hmC modification DNA, but not the 5mC modification DNA (10). All of these SRA domain-containing endonucleases cleave DNA at a certain distance away from the modified cytosine (10). In addition, SRA domains belong to the PUA superfamily that includes other modified DNA/RNA-binding domains, such as EVE domains and YTH domains (2, 12, 13). These domains can also be fused to endonucleases, and most of the fusion proteins function as cytosine-modification-specific endonucleases (2, 12). Compared to the characterized cytosine modification-dependent endonucleases, very few m6A-dependent restriction endonucleases have been identified, and DpnI is the best-known example (2, [Bibr B14][Bibr B15][Bibr B17]). The catalytic properties of DpnI are distinct from those of the SRA domain-containing MDREs. DpnI recognizes the Gm6ATC sites and efficiently cleaves fully methylated DNA, leaving a blunt end, while it also cleaves hemi-methylated DNA to a lesser extent (15). DpnI consists of an N-terminal PD-(D/E)XK nuclease domain and a C-terminal winged helix (wH) domain of the helix-turn-helix fold family, and both domains determine the specificity of the recognition sequence (3, 15).

Restriction endonucleases are ubiquitously encoded in prokaryotes. Exceptions are restriction endonucleases found in some chlorella viruses and giant viruses (18 to 21). Unlike the antiviral functions of restriction endonucleases present in prokaryotes, the viral enzymes may contribute to degrading host genomes to recycle deoxynucleotides for viral DNA replication or to exclude other viruses in the cases of multiple infections (21, 22). However, to date, no MDREs have been found in viruses. In this study, we describe a unique MDRE (HHPV4I) from an archaeal virus HHPV4 (Haloarcula hispanica pleomorphic virus 4). Interestingly, HHPV4 is the only described member that encodes an MDRE in the family *Pleolipoviridae* (23, 24). HHPV4I consists of an N-terminal SRA domain variant, a middle wH domain, and a C-terminal HNH domain (an endonuclease of SRA-wH-HNH type). As a unique m6A-dependent endonuclease, the catalytic properties of HHPV4I are distinct from those of DpnI and other characterized SRA-containing endonucleases.

## RESULTS

### A site-specific endonuclease identified only in archaeal virus HHPV4.

**FIG 1:**
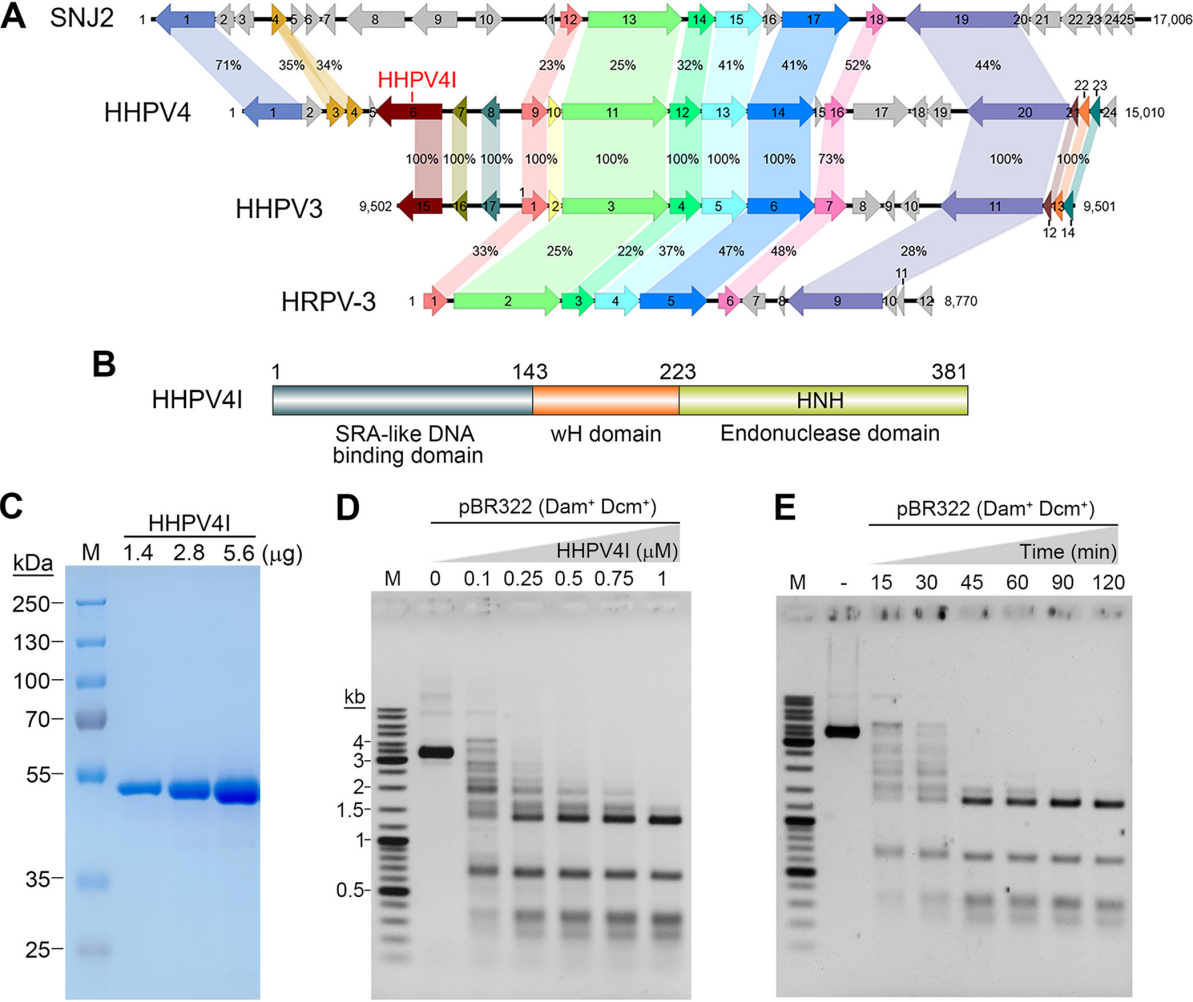
Identification of a special endonuclease (HHPV4I) from HHPV4. (A) Comparison of the genomic organization of HHPV4, SNJ2, HHPV3, and HRPV-3. These viruses have similar genomic organization, while a putative endonuclease (HHPV4I) is only encoded in HHPV4. (B) The domain architecture of HHPV4I. HHPV4I contains three domains: an SRA domain, a wH domain, and an HNH domain. (C) SDS-PAGE analysis of the purified HHPV4I. (D) Plasmid pBR322 was digested by various concentrations of HHPV4I for 1 h. (E) Plasmid pBR322 was digested by 0.5 μM HHPV4I for different lengths of time.

HHPV4 is an archaeal virus to be isolated and characterized that infects the extremely halophilic archaeon H. hispanica (23). The virus has a circular double-stranded (ds) DNA genome and belongs to the genus *Betapleolipovirus* of the archaeal virus family *Pleolipoviridae* (23, 24), which also includes the previously characterized viruses SNJ2 (Saline *Natrinema* sp. J7-1 virus 2) (25), HHPV3 (Haloarcula hispanica pleomorphic virus 3) (26), and HRPV-3 (*Halorubrum* sp. pleomorphic virus 3) (27). Visualization of the viral genomes (Fig. 1A) was performed with Easyfig (28). Comparison of the genome organizations of HHPV4, SNJ2, HHPV3, and HRPV-3 showed that these virus genomes shared a cluster of conserved genes (Fig. 1A). Notably for HHPV4 and HHPV3, about 87% of the HHPV3 genome sequence was identical to the HHPV4 genome sequence, and 12 of 17 putative proteins encoded in HHPV3 were 100% identical to the putative proteins encoded in HHPV4 (Fig. 1A), showing the high homology between the two viruses. However, by analyzing putative proteins encoded in HHPV4, we found that the gene 6 encodes a special endonuclease that is not present in other pleolipoviruses (Fig. 1A). This predicted endonuclease (denoted as HHPV4I) is composed of three domains: an N-terminal SRA domain variant, a middle wH domain, and a C-terminal HNH domain (Fig. 1B). Interestingly, the gene 15 of HHPV3 encodes a putative protein, part of which shows 100% amino acid sequence identity with the HHPV4I, but the protein lacks the potential catalytic HNH domain (Fig. 1A).

To study the catalytic properties of HHPV4I, we constructed the HHPV4I expression plasmid and overexpressed and purified HHPV4I (Fig. 1C). To verify the cleavage activity of HHPV4I, we performed experiments to test whether HHPV4I could cleave plasmid pBR322. We first tested the cleavage efficiency of different HHPV4I concentrations to the plasmid pBR322. The results showed that plasmid pBR322 could be cleaved into several small fragments in the presence of 0.1 μM HHPV4I, and the lengths of the cleaved fragments decreased with increasing concentrations of HHPV4I (Fig. 1D). Four specific DNA fragments could be observed in the presence of 1 μM HHPV4I (Fig. 1D), demonstrating that HHPV4I is a site-specific endonuclease. Time-course analysis of the HHPV4I activity showed that the four specific DNA fragments could not be further cleaved during prolonged incubation (Fig. 1E), further confirming the cleavage specificity of HHPV4I.

### HHPV4I is a Mn^2+^- and m6A-dependent DNA endonuclease.

**FIG 2:**
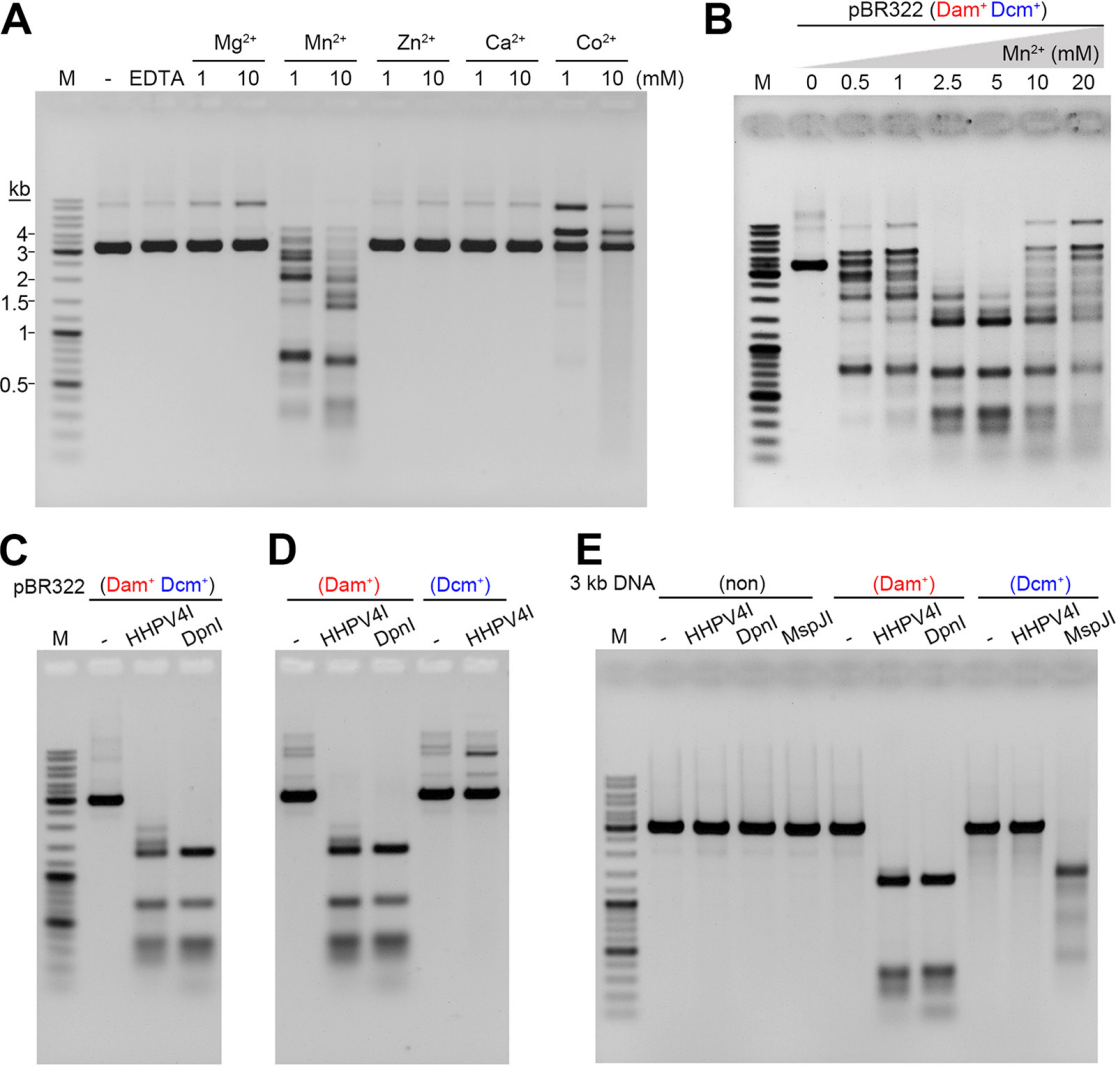
Effects of divalent cations and DNA methylation on the cleavage activity of HHPV4I. (A) Effects of different divalent cations on the cleavage activity of HHPV4I. Plasmid pBR322 can only be efficiently cleaved by HHPV4I in the presence of Mn^2+^. (B) Effects of different concentrations of Mn^2+^ on the cleavage activity of HHPV4I. The optimal concentration of Mn^2+^ for HHPV4I cleavage is 5 mM Mn^2+^. (C) The plasmid pBR322 isolated from E. coli DH5α was digested by HHPV4I or DpnI. (D) The plasmid pBR322 isolated from E. coli JM110 was methylated by Dam methylase or Dcm methylase *in vitro* and then digested by HHPV4I or DpnI. (E) 3-kb PCR product (F1) was nonmethylated or was methylated by Dam methylase or Dcm methylase *in vitro* and then digested by HHPV4I, DpnI, or MspJI.

We next investigated the effects of different divalent cations on the cleavage efficiency of HHPV4I. Using plasmid pBR322 as the substrate, the cleavage activity of HHPV4I was examined in the presence of Mg^2+^, Mn^2+^, Zn^2+^, Ca^2+^, or Co^2+^ at concentrations of 1 mM and 10 mM. The results showed that the plasmid pBR322 could only be efficiently cleaved into small fragments in the presence of Mn^2+^ (Fig. 2A), suggesting that HHPV4I is a Mn^2+^-dependent endonuclease. Next, we examined HHPV4I cleavage activity in the presence of various concentrations of Mn^2+^. The results showed that 5 mM Mn^2+^ in the reaction mixture was optimal for HHPV4I activity (Fig. 2B). Thus, the next experiments were performed in the presence of 5 mM Mn^2+^.

Because HHPV4I was shown to be a site-specific endonuclease, we next wanted to determine the recognition sequence of HHPV4I. As the plasmid pBR322 that we had used was isolated from E. coli DH5α with 5mC modifications at the C5mCWGG sites and m6A modifications at the Gm6ATC sites, this plasmid can be cleaved by DpnI at the Gm6ATC site. Interestingly, using DpnI as a control, we found that the sizes of the fragments cleaved by HHPV4I were similar to the sizes of the fragments cleaved by DpnI (Fig. 2C), indicating that HHPV4I may also recognize the Gm6ATC site. To further identify the DNA modification recognized by HHPV4I, we isolated the nonmethylated plasmid pBR322 from E. coli JM110 that lacked Dam methyltransferase and Dcm methyltransferase. The nonmethylated plasmid pBR322 was methylated by Dam methyltransferase or Dcm methyltransferase *in vitro*. Indeed, only Dam-methylated plasmid pBR322 could be cleaved by HHPV4I as well as DpnI (Fig. 2D). Next, a 3-kb DNA fragment (F1; Table S1 in the supplemental material), which was obtained by PCR from the plasmid pBR322, was used to further verify the recognition site of HHPV4I. The DNA F1 was methylated by Dam methyltransferase or Dcm methyltransferase *in vitro*, and then nonmethylated, Dam-methylated, and Dcm-methylated DNA F1 were used as the substrates. The results showed that HHPV4I only cleaved Dam-methylated DNA F1 but not nonmethylated or Dcm-methylated DNA F1 as DpnI, while MspJI (the 5mC-dependent endonuclease used as a control) only cleaved Dcm-methylated DNA F1 but not nonmethylated or Dam-methylated DNA F1 (Fig. 2E). These results demonstrated that HHPV4I is an m6A-dependent DNA endonuclease that recognizes the Gm6ATC site.

### The wH and HNH domains, but not the SRA domain, are responsible for m6A-dependent endonuclease activity.

**FIG 3:**
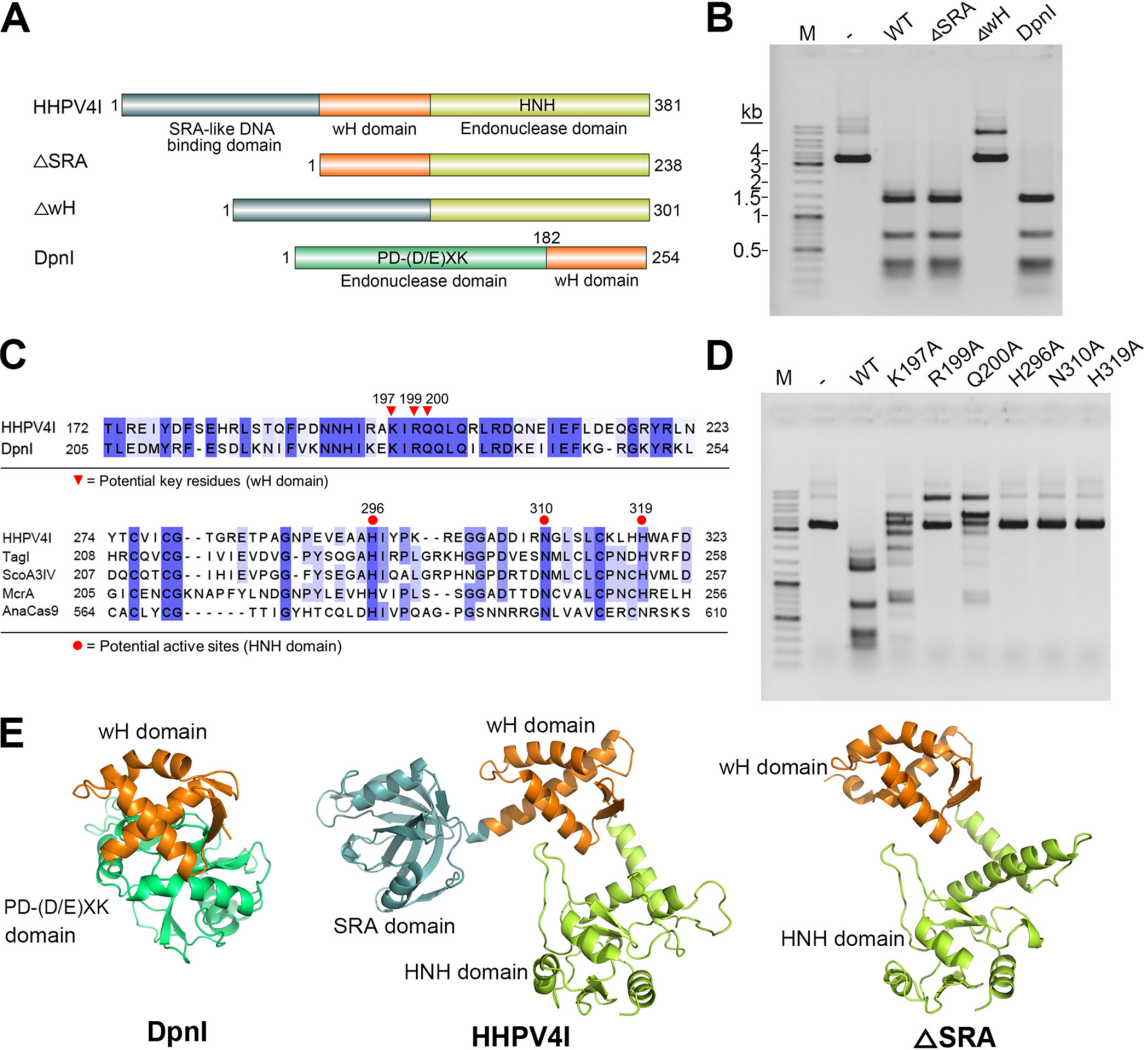
Comparison of the cleavage activities of HHPV4I, its domain deletion mutants, and its single-residue mutants. (A) Schematic diagram of the domain architectures of HHPV4I mutants and DpnI. (B) Plasmid pBR322 was digested by HHPV4I, ΔSRA, ΔwH, or DpnI. (C) Potential key residues in the wH domain and potential active site residues in the HNH domain were identified based on sequence alignment of the wH domains of HHPV4I and DpnI and sequence alignment of the HNH domains of HHPV4I and other HNH endonucleases. (D) Plasmid pBR322 was digested by HHPV4I (WT) or its single-residue mutants. (E) RoseTTAFold structural predictions of DpnI, HHPV4I, and ΔSRA.

As mentioned above, HHPV4I contains three domains: an SRA domain, a wH domain, and an HNH domain (Fig. 1B). To investigate the role of each domain in the HHPV4I cleavage activity, we constructed domain deletion mutants that lacked the SRA domain (ΔSRA) or wH domain (ΔwH) (Fig. 3A and Fig. S1). We also tried to express and purify the HNH domain deletion mutant (ΔHNH); however, the expression level of ΔHNH was extremely low and we could hardly obtain purified ΔHNH mutant. The cleavage activities of the mutants were examined using the plasmid pBR322 as the substrate. Compared to the wild-type (WT) HHPV4I and DpnI, ΔwH was unable to cleave plasmid pBR322 into small fragments, while ΔSRA retained specific cleavage activity (Fig. 3B), suggesting that the wH domain but not the SRA domain is responsible for the HHPV4I activity. Interestingly, DpnI also contains a wH domain that exhibits homology with the HHPV4I wH domain (Fig. 3A). A previous study has shown that the wH domain of DpnI is crucial for its activity, and the key residues (K229, R231, and Q235) in the DpnI wH domain have been identified (15). To identify the potential key residues in the HHPV4I wH domain, we aligned the HHPV4I wH domain sequences and the DpnI wH domain sequences and identified the corresponding key residues (K197, R199, and Q200) in the wH domain of HHPV4I (Fig. 3C). We then constructed the corresponding mutants (K197A, R199A, and Q200A) and examined their cleavage activities (Fig. S1). Compared to HHPV4I (WT), all of the wH domain mutants exhibited lower cleavage activities, especially the R199A, which only exhibited weak nicking activity (Fig. 3D). The results showed that these residues are crucial for HHPV4I activity and further demonstrated that the wH domain is crucial for HHPV4I activity. In addition, we also aligned the HHPV4I HNH domain and some other HNH domains of endonucleases and identified the key residues (H296, N310, and H319) in the HNH domain of HHPV4I (Fig. 3C). We then constructed the corresponding mutants (H296A, N310A, and H319A) and examined their activities (Fig. 3D and Fig. S1). As expected, these mutants completely lost the cleavage activity (Fig. 3D), demonstrating the critical role of the HNH domain for HHPV4I cleavage activity. In addition, the predicted structures showed that the HHPV4I wH domain adopts a fold similar to the DpnI wH domain, further suggesting the crucial role of the wH domain for Gm6ATC site recognition (Fig. 3E). Interestingly, SRA domain deletion had almost no effect on the wH domain conformation of HHPV4I, while the deletion affected the local conformation of the HHPV4I HNH domain (Fig. 3E).

### Both the wH and HNH domains of HHPV4I are responsible for binding the recognition site.

**FIG 4:**
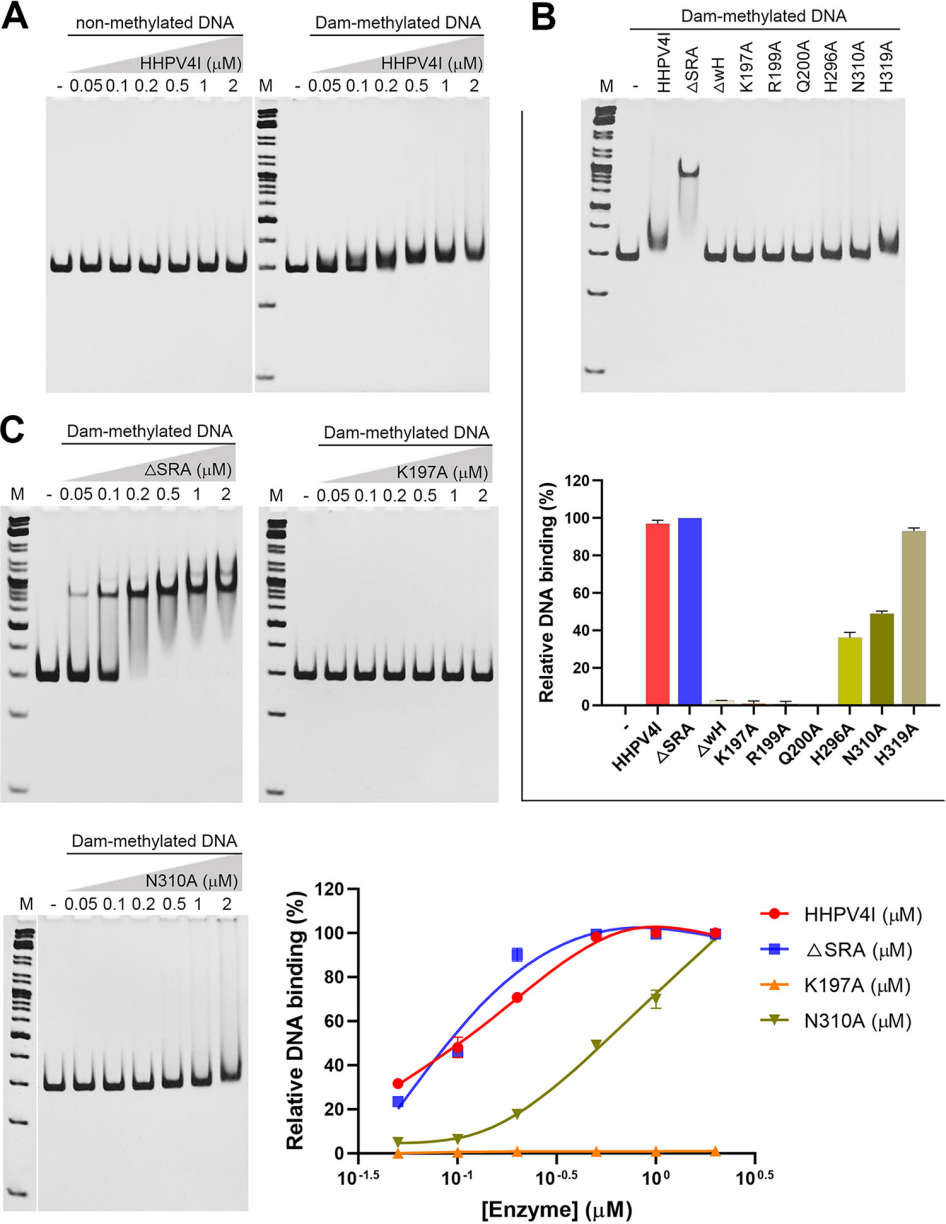
The binding affinity of HHPV4I and its mutants to the Dam-methylated DNA. (A) Comparison of the binding affinity of HHPV4I to the nonmethylated DNA and Dam-methylated DNA. A 300-bp PCR product (F2) containing three GATC sites was methylated by Dam methylase *in vitro*. The DNA binding activity of HHPV4I was evaluated by EMSA. (B) Comparison of the binding affinity of HHPV4I and its mutants to the Dam-methylated DNA (F2). (C) Evaluation of the DNA binding activity of different mutants (△SRA, K197A, and N310A) with different concentrations. Experimental data are representative of two independent experiments.

A previous study has shown that both the PD-(D/E)XK domain and the wH domain of DpnI showed binding affinity to the recognition site Gm6ATC (15). Our results have shown that HHPV4I also contains a wH domain with high homology to the DpnI wH domain, which is crucial for HHPV4I activity (Fig. 3C, D). To explore whether the HHPV4I or its mutants could specifically bind to the Gm6ATC site, we performed an electrophoretic mobility shift assay (EMSA) using nonmethylated or Dam-methylated 300 bp DNA (F2; Table S1). The results showed that HHPV4I only showed binding affinity to the Dam-methylated DNA but not the nonmethylated DNA, suggesting that HHPV4I specifically binds to DNA containing the Gm6ATC site (Fig. 4A). Next, we tested the binding activities of different HHPV4I mutants. Compared to HHPV4I (WT), ΔSRA showed tight binding to the Dam-methylated DNA, whereas ΔwH showed almost no detectable binding activity for the Dam-methylated DNA, suggesting that the wH domain but not the SRA domain is important for DNA binding (Fig. 4B). The single-residue mutants K197A, R199A, and Q200A showed almost no detectable binding activity for the Dam-methylated DNA (Fig. 4B), further demonstrating that the key residues K197, R199, and Q200 in the wH domain are responsible for binding the recognition site Gm6ATC. The defects of these wH domain mutants on recognition site binding should be responsible for their impaired DNA cleavage activity (Fig. 3D). Interestingly, single-residue mutants H296A and N310A also showed reduced binding activities (Fig. 4B), suggesting that the HHPV4I HNH domain also contributes to the binding of recognition site Gm6ATC. Next, we also evaluated the DNA binding activities of mutants (ΔSRA, K197A, and N310A) with different concentrations. The results showed that ΔSRA actually has a higher DNA binding activity than the wild-type HHPV4I; N310A showed a lower DNA binding activity than HHPV4I, whereas K197A showed almost no detectable DNA binding activity (Fig. 4C), further demonstrating that both the wH domain and the HNH domain are responsible for the binding to Gm6ATC site-containing DNA. The conformational changes of the HNH domain resulting from SRA domain deletion (Fig. 3E) may explain the enhanced DNA binding activity of ΔSRA.

### The cleavage pattern of HHPV4I is distinct from that of DpnI.

**FIG 5:**
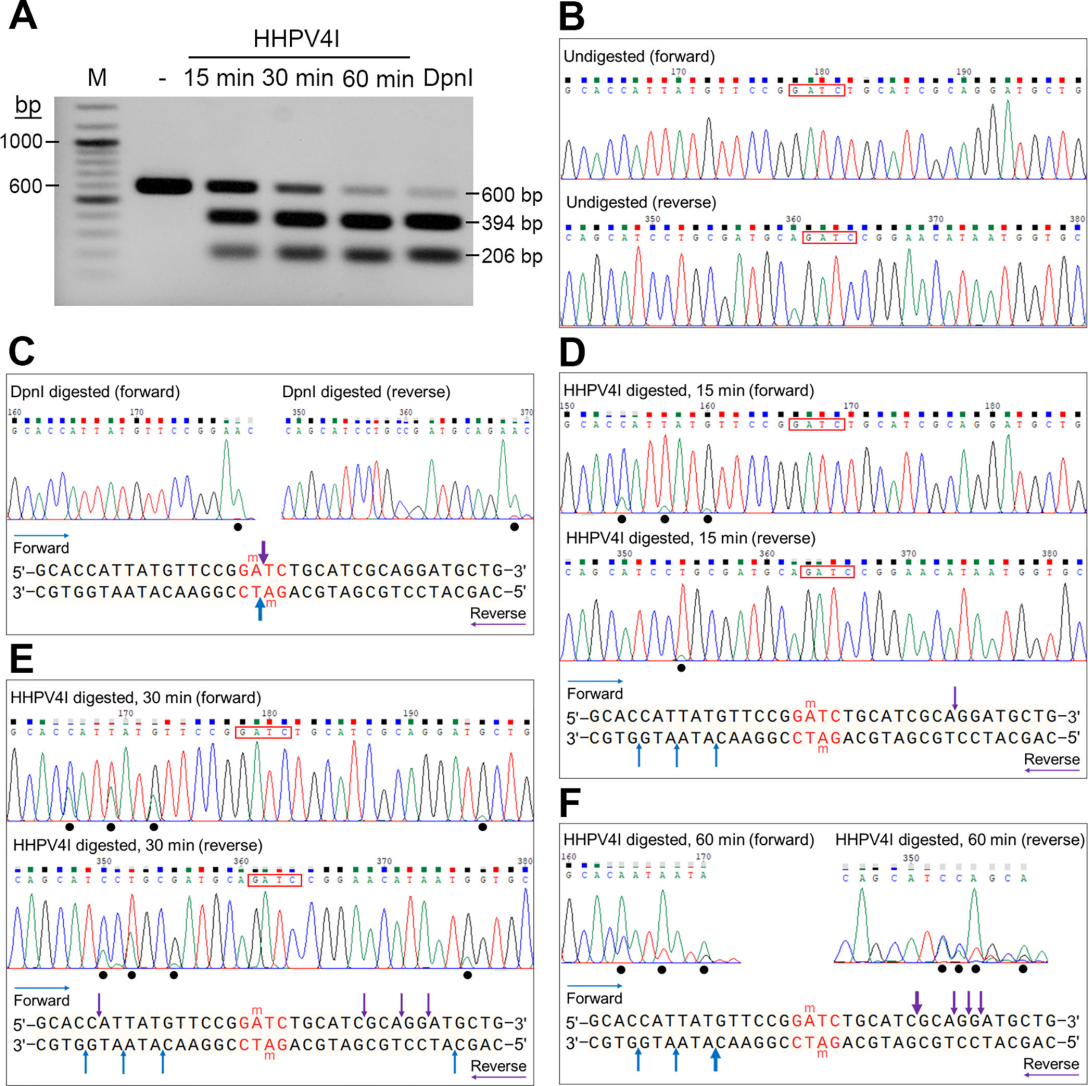
Determination of the HHPV4I-generated cleavage sites by runoff sequencing. (A) Agarose gel showing the Dam-methylated DNA F3 digested by HHPV4I for 15 min, 30 min, and 60 min, with DpnI used as a control. (B) The Dam-methylated DNA F3 without HHPV4I digestion was sequenced by runoff sequencing as a negative control. (C) Dam-methylated DNA F3 was digested by DpnI, and then the generated cleavage sites were determined by using runoff sequencing. (D to F) The Dam-methylated DNA F3 were digested by HHPV4I for 15 min (D), 30 min (E), and 60 min (F), and then the generated cleavage sites were determined by using runoff sequencing.

Our results had shown that HHPV4I cleaves DNA containing the Gm6ATC site as DpnI, although it was unknown whether the cleavage manner of HHPV4I was similar to that of DpnI. To further explore the cleavage properties of HHPV4I, a Dam-methylated PCR fragment (F3; Table S1) containing only one Gm6ATC site was digested by HHPV4I for different lengths of time. Agarose gel electrophoresis showed that the amounts of the cleavage products increased with increasing cleavage time, and their sizes corresponded to the sizes of DpnI-cleaved products (Fig. 5A). Next, we determined the cleavage sites by runoff sequencing. As expected, the sequencing results showed that no cleavage site could be observed in the undigested DNA (Fig. 5B), and DpnI cleaves DNA at the recognition site Gm6A|TC (where | indicates the cleavage site) (Fig. 5C). Unexpectedly, the DNA cleavage sites generated by HHPV4I were outside the recognition site Gm6ATC (Fig. 5D to F). The cleavage sites were observed downstream of the Gm6ATC sites of both DNA strands at the digestion time of 15 min (Fig. 5D) and both downstream and upstream of the Gm6ATC sites at the digestion time of 30 min (Fig. 5E). The cleavage sites were only detected downstream of the Gm6ATC sites at the digestion time of 60 min (Fig. 5F), suggesting that the DNA had been almost completely cleaved at that time. The results showed that HHPV4I exhibits a cleavage pattern that is distinct from that of DpnI. In addition, the Dam-methylated DNA F3 was also digested by ΔSRA for different lengths of time, and the cleavage sites were determined by runoff sequencing (Fig. S2). The results showed that the cleavage sites generated by ΔSRA at different lengths of time were similar to those generated by HHPV4I, indicating that SRA deletion does not alter the cleavage specificity of HHPV4I.

**FIG 6:**
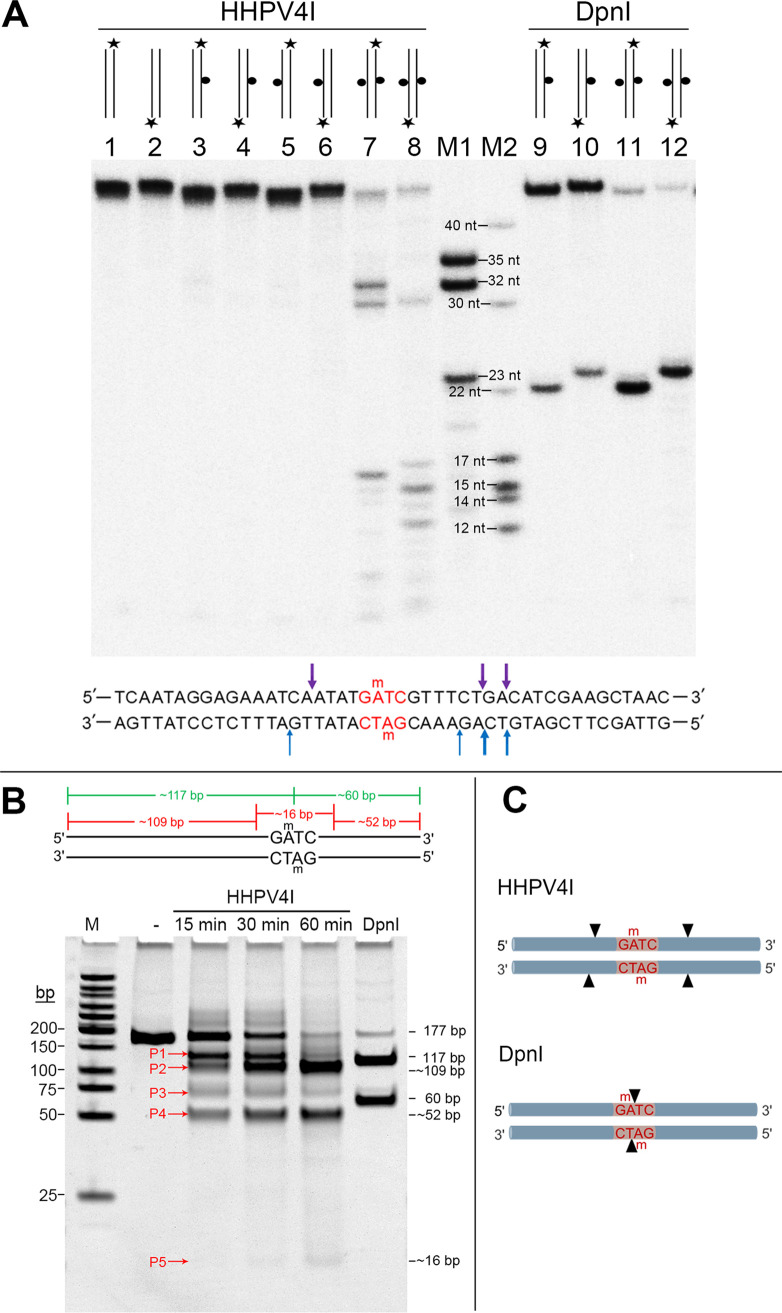
Comparison of the cleavage patterns of HHPV4I and DpnI. (A) Denaturing urea PAGE analysis of the products of the 45-bp oligoduplexes cleaved by HHPV4I and DpnI. The radiolabeled strands are represented by black asterisks, and the m6A modification in the GATC sites is represented by black dots in the diagram above each lane. M1 and M2 represent the DNA markers. The reactions contained 50 nM of the indicated oligoduplexes and 500 nM HHPV4I and were incubated for 1 h at 37°C. The reaction products were separated on a 7-M urea-10% PAGE. (B) Nondenaturing acrylamide gel analysis of the products of the designed 177-bp DNA cleaved by HHPV4I and DpnI. The 177-bp DNA (F4) was methylated by Dam methylase *in vitro*, and then was digested by HHPV4I for different lengths of time. P1 and P4 indicate the partial cleavage products, and P2, P3, and P5 indicate the final cleavage products. (C) Schematic diagram of the different cleavage sites generated by HHPV4I and DpnI.

To further determine the cleavage pattern of HHPV4I, we designed a set of 45-bp oligoduplex substrates to test HHPV4I cleavage activity. The oligoduplexes containing a nonmethylated or m6A-methylated GATC site in the top strand, bottom strand, or both strands were employed in a cleavage assay. As expected, DpnI could efficiently cleave the designed oligoduplexes with fully methylated Gm6ATC sites to generate 22-nt and 23-nt fragments, and it could also cleave the designed oligoduplexes with hemi-methylated Gm6ATC sites to a lesser extent (Fig. 6A, lanes 9 to 12). In contrast, HHPV4I could hardly cleave the oligoduplexes with hemi-methylated Gm6ATC sites (Fig. 6A, lanes 3 to 6). As a control, HHPV4I was unable to cleave the oligoduplexes with nonmethylated GATC sites, whereas it efficiently cleaved the oligoduplexes with fully methylated Gm6ATC sites (Fig. 6A, lanes 1 to 2, 7 to 8). However, the HHPV4I-cleaved products were not as fixed as DpnI-cleaved products (Fig. 6A, lanes 7 to 8, 11 to 12). According to the sizes of the cleavage products, we confirmed that the cleavage sites generated by HHPV4I were either upstream or downstream of the recognition site Gm6ATC (Fig. 6A), consistent with the results observed by runoff sequencing (Fig. 5). Because HHPV4I cleaves DNA at both upstream and downstream sites of the recognition site Gm6ATC, theoretically the DNA will be eventually cleaved into three fragments, of which a very small fragment will contain the Gm6ATC site. To directly visualize the HHPV4I-cleaved fragments, we next designed a 177-bp DNA (F4; Table S1) that contained one Gm6ATC site and that could be cleaved by DpnI to generate a 117-bp fragment and a 60-bp fragment. The HHPV4I-cleaved and DpnI-cleaved products were separated by nondenaturing PAGE, and then the gel was stained with ethidium bromide (EB) to visualize each cleaved fragment in double-stranded form. The results clearly showed that the HHPV4I-cleaved products were different from the DpnI-cleaved products (Fig. 6B). Furthermore, as determined by the time course analysis, the amounts of the partial cleavage products (P1 and P3) decreased with increasing cleavage time, while the amounts of the final cleavage products (P2, P4, and P5) increased with increasing cleavage time (Fig. 6B). Consistent with the above analysis, a very small fragment (P5) could be clearly visualized on the gel at the reaction time of 1 h (Fig. 6B); this should be the fragment containing the Gm6ATC site. Based on the above results, we established a model for comparison of the cleavage patterns of HHPV4I and DpnI (Fig. 6C) that clearly shows the different cleavage sites generated by HHPV4I and DpnI.

### Characteristics of cleavage sites generated by HHPV4I.

**FIG 7:**
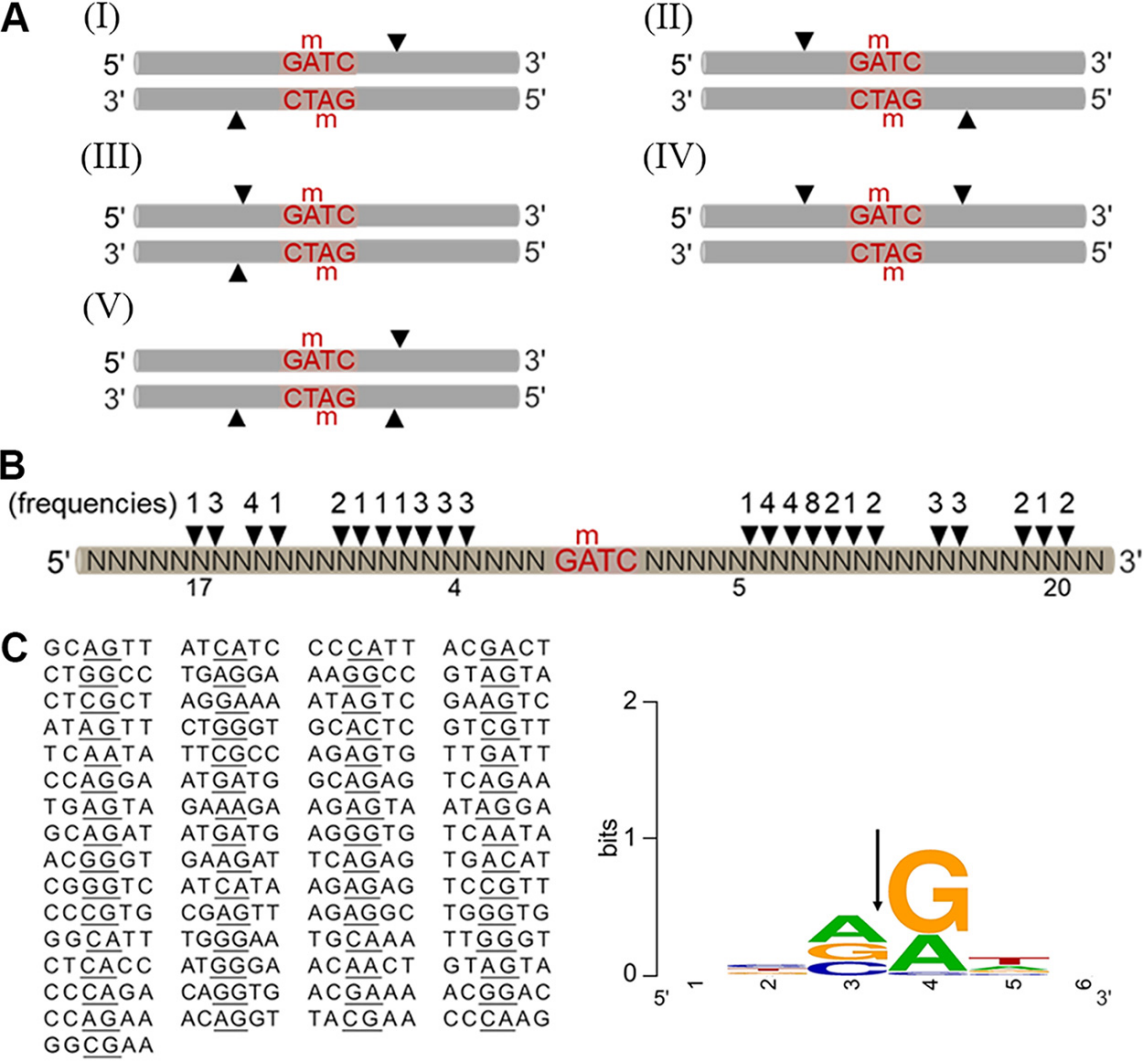
Characterization of cleavage sites generated by HHPV4I. (A) Summary of the process of HHPV4I digestion. Different Dam-methylated DNA fragments were partially digested by HHPV4I (digestion for 15 min), and then the products were sequenced by runoff sequencing to determine the cleavage sites. Arrowheads indicate the cleavage sites detected from different partial cleavage products. (B) Cartoon representation of the frequencies of the cleavage sites and the distances from the cleavage sites to the Gm6ATC sites. Dam-methylated DNA fragments were digested by HHPV4I for 30 min. The generated cleavage sites were determined by using runoff sequencing and were counted. (C) Mapping of the cleavage sites. The cleavage sites were summarized from Dam-methylated DNA fragments digested by HHPV4I for 30 min. The sequence logo was generated using WebLogo (35). The arrow denotes the site of DNA nicking.

Partial cleavage products were generated during HHPV4I digestion (Fig. 5 and 6B), indicating that HHPV4I does not simultaneously cleave upstream and downstream locations of Gm6ATC sites on both DNA strands. To determine the cleavage process of HHPV4I, we systematically examined the partial cleavage products generated by HHPV4I digestion for a short time (15 min). Ten different DNA fragments containing a GATC site were methylated by Dam *in vitro* and then were subjected to HHPV4I digestion (Fig. S3). As shown in Fig. 7A, the cleavage sites were detected only downstream of the Gm6ATC sites on both DNA strands (I), only upstream of the Gm6ATC sites on both DNA strands (II), upstream of the Gm6ATC site on one DNA strand and downstream of the Gm6ATC site on the other DNA strand (III), both upstream and downstream of the Gm6ATC site on one DNA strand (IV), or both upstream and downstream of the Gm6ATC site on one DNA strand and downstream of the Gm6ATC site on the other DNA strand (V). These detected cleavage sites suggest that the cleavage processes for different DNA fragments are variable and that the DNA sequence contexts around the Gm6ATC sites may affect the cleavage process of HHPV4I. In addition, the results also suggest that HHPV4I may independently nick DNA upstream and downstream of the Gm6ATC sites on both DNA strands.

Moreover, we also observed that distances from the cleavage sites to the Gm6ATC site were not fixed. To detail the positions of the cleavage sites, different Dam-methylated DNA fragments were cleaved by HHPV4I for 30 min (Fig. S4), and the positions of the cleavage sites were counted. As shown in Fig. 7B, positions of the cleavage sites were variable, 4 to 20 nt away from the Gm6ATC site. Interestingly, HHPV4I apparently prefers to cleave DNA between VR bases (V = A/G/C, R = A/G) (Fig. 7C).

### HHPV4I wH domain mutation K197A alters the sequence recognition specificity.

**FIG 8:**
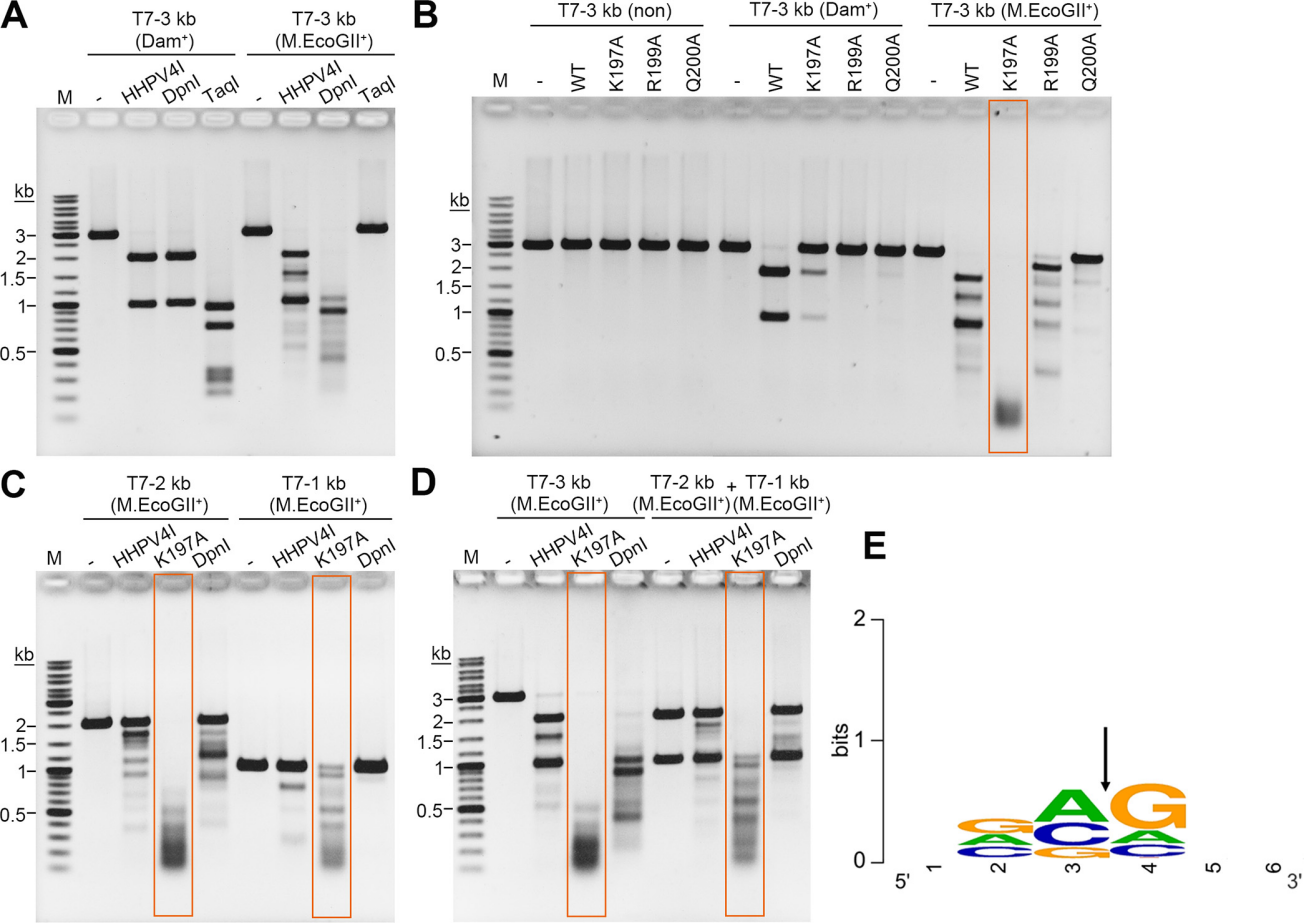
Mutant K197A cleaves diverse m6A-containing DNA sequences. (A) Comparison of the cleavage activities of HHPV4I and DpnI on the Dam-methylated DNA and M.EcoGII-methylated DNA. A 3-kb DNA fragment containing one GATC site was amplified from the T7 phage genome (T7-3 kb) and was methylated by Dam methylase or M.EcoGII. The methylated DNA fragment was then subjected to HHPV4I and DpnI digestion for 2 h. Endonuclease TaqI was used as a control, as the enzyme can cleave Dam-methylated DNA but not the M.EcoGII-methylated DNA. (B) Comparison of the cleavage activities of HHPV4I (WT) and wH domain mutants (K197A, R199A, and Q200A) on the nonmethylated, Dam-methylated, or M. EcoGII-methylated DNA. The methylated DNA was digested by WT and wH domain mutants for 2 h. (C) Comparison of the cleavage activities of HHPV4I, DpnI, and K197A on the M.EcoGII-methylated T7-2 kb DNA and T7-1 kb DNA. The T7-3 kb DNA fragment was divided into a 2-kb DNA fragment (T7-2 kb) and a 1-kb DNA fragment (T7-1 kb) from the GATC site by PCR amplification. The DNA fragments were methylated by M.EcoGII and then were subjected to digestion by HHPV4I, DpnI, and K197A for 2 h. (D) Comparison of the cleavage activities of HHPV4I, DpnI, and K197A on the M.EcoGII-methylated T7-3 kb DNA and T7-2 kb DNA plus T7-1 kb DNA. The methylated DNA was digested by HHPV4I and DpnI for 1 h. (E) Mapping of the cleavage sites of K197A. M.EcoGII-methylated T7-1 kb DNA was digested by K197A for 30 min. The cleavage sites were summarized, and the sequence logo was generated using WebLogo (35). The arrow denotes the site of DNA nicking.

We next sought to investigate whether HHPV4I could recognize other m6A modification sites. A 3-kb DNA fragment (T7-3 kb; Table S1) containing one GATC site was methylated by Dam methyltransferase or M.EcoGII, a nonspecific adenine DNA methyltransferase (29). As expected, both HHPV4I and DpnI cleaved the Dam-methylated DNA to generate two observable fragments (~2 kb and ~1 kb) (Fig. 8A). Using the M.EcoGII-methylated DNA fragment as a substrate, HHPV4I weakly cleaved DNA at other sites (Fig. 8A). Similarly, DpnI also cleaved DNA at other sites (Fig. 8A). As we had demonstrated that the HHPV4I wH domain is responsible for Gm6ATC site recognition, we next sought to investigate whether mutations in the wH domain could alter sequence recognition specificity of HHPV4I. As expected, HHPV4I and wH domain mutants (K197A, R199A, and Q200A) were unable to cleave nonmethylated T7-3 kb DNA; HHPV4I efficiently cleaved Dam-methylated T7-3 kb DNA; and K197A exhibited weak activity toward the Dam-methylated T7-3 kb DNA, whereas wH domain mutants R199A and Q200A showed almost no activity toward the Dam-methylated T7-3 kb DNA (Fig. 8B). However, compared to HHPV4I, R199A, and Q200A, K197A was able to efficiently cleave M.EcoGII-methylated DNA into numerous small fragments (Fig. 8B), indicating that K197A can recognize various m6A-containing sequences. The T7-3 kb DNA was divided into two fragments (T7-2 kb and T7-1 kb, Table S1) from the GATC site by PCR amplification, and then the two fragments were methylated by M.EcoGII. Next, we evaluated the cleavage activity of K197A on the M.EcoGII-methylated T7-2 kb DNA and T7-1 kb DNA that do not contain the Gm6ATC site. Compared to HHPV4I and DpnI, K197A could efficiently cleave the two methylated DNA fragments (Fig. 8C), further demonstrating that K197A cleaves various m6A-containing sequences. We also noticed that K197A cleaved M.EcoGII-methylated T7-3 kb DNA more efficiently than M.EcoGII-methylated T7-2 kb DNA or M.EcoGII-methylated T7-1 kb DNA (Fig. 8B and C). We next compared the cleavage activities of HHPV4I, K197A, and DpnI on the M.EcoGII-methylated T7-3 kb DNA and M.EcoGII-methylated T7-2 kb DNA plus M.EcoGII-methylated T7-1 kb DNA. Interestingly, although the total amount of DNA for the digestion was the same, K197A cleaved M.EcoGII-methylated T7-3 kb DNA more efficiently than M.EcoGII-methylated T7-2 kb DNA plus M.EcoGII-methylated T7-1 kb DNA (Fig. 8D). It is likely that cleavage at one site will facilitate cleavage at other sites for HHPV4I, similar to the 5mC modification-dependent endonuclease TagI that was found to cleave DNA more efficiently when the substrate DNA contained more 5mC recognition sites (10). In addition, the results also suggest that the presence of the Gm6ATC site triggers DpnI cleavage at other sites (Fig. 8D). We next determined the cleavage sites generated by K197A digestion. M.EcoGII-methylated T7-1 kb DNA was digested by K197A for 30 min, and then the cleavage sites were determined by runoff sequencing (Fig. S5). A total of 92 cleavage site sequences were identified (Fig. S5). As shown in Fig. 8E, K197A prefers to cleave DNA at VV|V sites, different from the wild-type HHPV4I preference (Fig. 7C), indicating that wH domain mutation also alters the preference of cleavage site.

## DISCUSSION

All of the previously identified and characterized MDREs were from prokaryotes, and they generally target viral genomes that contain modified cytosine bases. HHPV4I, an m6A-dependent endonuclease, is the first MDRE characterized from a virus. Archaeal virus HHPV4 has a genomic organization similar to that of other viruses in the genus *Betapleolipovirus*, but only the genome of HHPV4 encodes a MDRE, HHPV4I. Actually, we used position-specific iterative (PSI)-BLAST to search for HHPV4I homologs and found no HHPV4I homologous protein present in any other viruses. However, a few HHPV4I homologs are found in halophilic archaeons with high amino acid sequence identity (58 to 80%), indicating that the HHPV4I gene may have been acquired by HHPV4 via horizontal transfer from a halophilic archaeon. HHPV4I encoded by HHPV4 may be involved in inhibition of multiplication of other viruses. The HHPV4 genome encodes an integrase (gene 1 product), suggesting that the viral genome can be integrated into the host cell genome. In this context, HHPV4 will confer immunity to the host against the viruses whose genomes contain the m6A modification at the GATC site. Indeed, many antiphage systems have recently been identified in the prophage genomes (30). These systems provide immune protection for their host against other viruses and indirectly promote fitness of the prophages (30). In addition, several viruses have recently been found to encode CRISPR-Cas systems or mini-CRISPR arrays to inhibit replication of other viruses (31, 32). These cases suggest that viruses have evolved to utilize various strategies to exclude other viruses in the cases of multiple infections, and here MDRE encoded by HHPV4 is a new example.

HHPV4I cleaves Gm6ATC site-containing DNA similar to DpnI, while the cleavage pattern of HHPV4I is distinct from the DpnI cleavage pattern. DpnI acts as a monomer and introduces dsDNA breaks at the recognition sites by sequential nicking, leaving blunt ends (15), while HHPV4I cleaves DNA both upstream and downstream of the Gm6ATC site, 4 to 20 bp away from the Gm6ATC site. In addition, HHPV4I prefers to cleave between VR bases. The results suggest that HHPV4I cleavage requires a specific recognition sequence as well as a specific cleavage site sequence. To our knowledge, the cleavage pattern of HHPV4I is not similar to that of any other characterized endonuclease.

HHPV4I has an SRA domain at its N terminus. The SRA domain is considered to bind to cytosine modifications of DNA, and many SRA domain-containing endonucleases have been characterized to be 5mC-, 5hmC-, or g5hmC-dependent endonucleases (7, 9 to 11). However, the SRA domain of HHPV4I seems to be inactivated. HHPV4I cannot cleave Dcm-methylated DNA, and the SRA domain is also not responsible for the m6A-dependent endonuclease activity of HHPV4I (Fig. 2D and 3B). We further investigated in more detail whether HHPV4I could recognize a cytosine modification. To this end, 1.2-kb fragments with full 5mC, 5hmC, or g5hmC modification, and nonmodified fragments, were subjected to HHPV4I digestion under various reaction conditions. However, HHPV4I could not efficiently cleave any tested cytosine-modified DNA (Fig. S6). We also tested whether HHPV4I could bind to DNA with different cytosine modifications. No detectable binding activity of HHPV4I to the DNA fragments with the cytosine modification was observed (data not shown). It is likely that the SRA domain is inactive. However, as HHPV3 encodes a protein (gene 15 product) containing an SRA domain with 100% amino acid identity to the SRA domain of HHPV4I, it is likely that this domain has a specific function not yet detected.

We noticed that HHPV4I has a wH domain homologous to the wH domain of DpnI. Both of the wH domains are responsible for binding to the recognition site. However, having only the wH domain is not sufficient, and both of the endonuclease domains of HHPV4I and DpnI are also involved in recognition site binding. HHPV4I has an HNH endonuclease domain, while DpnI has a PD-(D/E)XK endonuclease domain. The different endonuclease domains of HHPV4I and DpnI may determine that HHPV4I and DpnI bind to the recognition site in different manners and exhibit different cleavage patterns. The deletion mutant ΔSRA of HHPV4I has a cleavage activity comparable to that of the wild-type HHPV4I, suggesting that the wH domain fused to the HNH endonuclease domain is sufficient for the m6A-dependent endonuclease activity. Moreover, key residue mutations in the HNH domain also affect the binding capacity of HHPV4I, suggesting communication between the HNH domain and the wH domain (3). Another interesting finding is that the wH domain mutation alters the recognition sequence specificity while retaining the m6A-dependent endonuclease activity. It is possible to engineer the wH domain of HHPV4I as well as DpnI to generate m6A-dependent endonucleases with various recognition sequences that are potentially valuable for m6A-related epigenetic analysis (33).

## MATERIALS AND METHODS

### Plasmid construction.

The codon-optimized HHPV4I gene was ordered from GenScript (Nanjing, China) and cloned into a pQE82L vector with an N-terminal 6×His tag. HHPV4I mutants were constructed by whole-plasmid PCR. For methylating DNA *in vitro*, the Dam methylase gene and the Dcm methylase gene were amplified from the E. coli DH5α genome and were inserted into plasmid pET28a with an N-terminal 6×His tag. The 5mC-dependent restriction endonuclease MspJI used as a control was also prepared in this study. The codon-optimized MspJI gene was ordered from GenScript (Nanjing, China) and was also cloned into a pQE82L vector with an N-terminal 6×His tag.

### Protein expression and purification.

HHPV4I and its mutants and MspJI were expressed in E. coli JM110 (Dam^−^ and Dcm^−^). The protein-expression plasmid of HHPV4I or its mutants was transformed into E. coli JM110. The transformants were inoculated in LB medium and grown overnight at 37°C. The cultures were transferred into fresh LB medium at a ratio of approximately 1:100. When the OD_600_ reached approximately 1.0, IPTG (isopropyl-β-d-thiogalactopyranoside) was added at 0.5 mM, and the cultures were incubated at 25°C for 5 h. Then, the cells were harvested and resuspended in lysis buffer (20 mM Tris-HCl, pH 8.0, 300 mM NaCl, 20 mM imidazole) and lysed by sonication on ice. The recombinant proteins were purified using Ni-NTA resin (Qiagen) and a gravity column. The Ni-NTA resin was washed (using lysis buffer supplemented to 50 mM imidazole) and eluted (using lysis buffer supplemented to 200 mM imidazole), and the eluate was dialyzed twice at 4°C against a storage buffer (50 mM Tris-HCl, pH 7.5, 100 mM NaCl, 0.1 mM EDTA, 1 mM DTT, 0.1% TritonX-100, and 50% glycerol). Dam methylase and Dcm methylase were expressed and purified using a similar procedure as described above, except that the proteins were expressed in E. coli BL21(DE3) instead of E. coli JM110.

### Preparation of DNA substrates for cleavage assays.

Plasmid pBR322 (Dam^+^ and Dcm^+^) was isolated from E. coli DH5α, and plasmid pBR322 (Dam^−^ and Dcm^−^) was isolated from E. coli JM110. Plasmid pBR322 (Dam^+^) and pBR322 (Dcm^+^) were prepared by methylating plasmid pBR322 (Dam^−^ and Dcm^−^) using Dam methylase and Dcm methylase, respectively. Different DNA fragments used in this study were amplified from plasmid pBR322, the T7 phage genome, or λ DNA (New England Biolabs). The sequences of the DNA fragments are listed in Table S1. The fragments were methylated *in vitro* by Dam methylase, Dcm methylase, or M.EcoGII (New England Biolabs). The DNA methylation reactions contained 50 mM Tris-HCl (pH 7.5), 10 mM EDTA, 5 mM β-mercaptoethanol, 160 μM S-adenosyl-l-methionine (SAM), the indicated DNA fragment, and Dam methylase, Dcm methylase, or M.EcoGII. Unless otherwise stated, the reaction mixtures were incubated for 1 h at 37°C. The methylated DNA was purified using a PCR purification kit (Qiagen).

### Cleavage assays.

The DNA cleavage reactions were carried out in a 10-μL final volume containing 50 mM Tris-HCl, pH 7.9, 100 mM NaCl, and unless otherwise stated, 5 mM MnCl_2_, 100 ng/μL of the indicated DNA substrate, and 500 nM HHPV4I or its mutants. The reaction mixtures were incubated at 37°C for the indicated time and terminated by adding 2 μL of 6× loading dye containing 60 mM EDTA for native gel electrophoresis. DpnI as a control was purchased from Thermo Fisher Scientific. The DpnI reactions were performed according to the manufacturer’s instructions.

### Electrophoretic mobility shift assay (EMSA).

A 300-bp DNA fragment (F2; Table S1) containing three GATC sites used in the EMSA was amplified from plasmid pBR322 and then methylated by Dam methylase. Binding reactions (20 μL final volume) containing 50 nM nonmethylated DNA F2 or Dam-methylated DNA F2 and various amounts of HHPV4I or its mutants were performed in 1× reaction buffer (20 mM Tris-HCl, pH 8.0, 100 mM KCl) at 37°C for 5 min. Then, the reactions were mixed with 4 μL of 6× loading dye and examined by 6% nondenaturing PAGE in 0.5× TBE (Tris-borate-EDTA).

### Denaturing PAGE analysis of the HHPV4I cleavage activity.

The 45-nt top strand and bottom strand with or without the m6A modification in the GATC site were synthesized by Sangon Biotech (Shanghai, China). One of the DNA strands was labeled at the 5′ end using T4 polymerase kinase (New England Biolabs) and [γ-^32^P] ATP (Perkin Elmer) and then annealed with the complementary strand. The reaction mixtures (10 μL) containing 50 mM Tris-HCl (pH 7.9), 100 mM NaCl, 5 mM MnCl_2_, 50 nM 5′-labeled oligoduplexes, and 500 nM HHPV4I were incubated at 37°C for 1 h, and then the reactions were terminated by adding 5 μL of 95% formamide dye containing 20 mM EDTA. Reaction products were heated for 3 min at 90°C and then examined by 10% denaturing PAGE containing 7 M urea. The gels were analyzed by a phosphoimager.

### Determination of the DNA cleavage site.

Various DNA fragments containing a GATC site were obtained by PCR amplification (Table S1) and were methylated using Dam methylase. The Dam-methylated DNA fragments were digested with HHPV4I for different lengths of time. The digested DNA fragments were purified using a PCR purification kit (Qiagen) and then were subjected to runoff sequencing. In the sequencing, *Taq* DNA polymerase would add an extrinsic A at the position where the DNA template is cleaved (2). Thus, the double peaks (A/T, A/C, A/G) in the sequencing results indicate the cleavage sites.

### RoseTTAFold structural prediction.

The structures of DpnI, HHPV4I, and the mutant ΔSRA were predicted using RoseTTAFold (34). The amino acid sequences of the proteins were submitted to the Robetta server (https://robetta.bakerlab.org/submit.php). The model structure ranked first among the output model structures (Model 1) for each protein was collected for further analysis, and the model structures were analyzed using PyMOL.

## References

[1] Loenen WA, Raleigh EA. 2014. The other face of restriction: modification-dependent enzymes. Nucleic Acids Res 42:56–69. doi:10.1093/nar/gkt747. 23990325 PMC3874153

[2] Lutz T, Flodman K, Copelas A, Czapinska H, Mabuchi M, Fomenkov A, He X, Bochtler M, Xu S-Y. 2019. A protein architecture guided screen for modification dependent restriction endonucleases. Nucleic Acids Res 47:9761–9776. doi:10.1093/nar/gkz755. 31504772 PMC6765204

[3] Weigele P, Raleigh EA. 2016. Biosynthesis and function of modified bases in bacteria and their viruses. Chem Rev 116:12655–12687. doi:10.1021/acs.chemrev.6b00114. 27319741

[4] Unoki M, Nishidate T, Nakamura Y. 2004. ICBP90, an E2F-1 target, recruits HDAC1 and binds to methyl-CpG through its SRA domain. Oncogene 23:7601–7610. doi:10.1038/sj.onc.1208053. 15361834

[5] Arita K, Ariyoshi M, Tochio H, Nakamura Y, Shirakawa M. 2008. Recognition of hemi-methylated DNA by the SRA protein UHRF1 by a base-flipping mechanism. Nature 455:818–821. doi:10.1038/nature07249. 18772891

[6] Han T, Yamada-Mabuchi M, Zhao G, Li L, Liu G, Ou H-Y, Deng Z, Zheng Y, He X. 2015. Recognition and cleavage of 5-methylcytosine DNA by bacterial SRA-HNH proteins. Nucleic Acids Res 43:1147–1159. doi:10.1093/nar/gku1376. 25564526 PMC4333417

[7] Zheng Y, Cohen-Karni D, Xu D, Chin HG, Wilson G, Pradhan S, Roberts RJ. 2010. A unique family of Mrr-like modification-dependent restriction endonucleases. Nucleic Acids Res 38:5527–5534. doi:10.1093/nar/gkq327. 20444879 PMC2938202

[8] Horton JR, Mabuchi MY, Cohen-Karni D, Zhang X, Griggs RM, Samaranayake M, Roberts RJ, Zheng Y, Cheng X. 2012. Structure and cleavage activity of the tetrameric MspJI DNA modification-dependent restriction endonuclease. Nucleic Acids Res 40:9763–9773. doi:10.1093/nar/gks719. 22848107 PMC3479186

[9] Cohen-Karni D, Xu D, Apone L, Fomenkov A, Sun Z, Davis PJ, Kinney SRM, Yamada-Mabuchi M, Xu S-y, Davis T, Pradhan S, Roberts RJ, Zheng Y. 2011. The MspJI family of modification-dependent restriction endonucleases for epigenetic studies. Proc Natl Acad Sci USA 108:11040–11045. doi:10.1073/pnas.1018448108. 21690366 PMC3131316

[10] Kisiala M, Copelas A, Czapinska H, Xu SY, Bochtler M. 2018. Crystal structure of the modification-dependent SRA-HNH endonuclease TagI. Nucleic Acids Res 46:10489–10503. doi:10.1093/nar/gky781. 30202937 PMC6212794

[11] Wang H, Guan S, Quimby A, Cohen-Karni D, Pradhan S, Wilson G, Roberts RJ, Zhu Z, Zheng Y. 2011. Comparative characterization of the PvuRts1I family of restriction enzymes and their application in mapping genomic 5-hydroxymethylcytosine. Nucleic Acids Res 39:9294–9305. doi:10.1093/nar/gkr607. 21813453 PMC3241641

[12] Pastor M, Czapinska H, Helbrecht I, Krakowska K, Lutz T, Xu S-Y, Bochtler M. 2021. Crystal structures of the EVE-HNH endonuclease VcaM4I in the presence and absence of DNA. Nucleic Acids Res 49:1708–1723. doi:10.1093/nar/gkaa1218. 33450012 PMC7897488

[13] Hosford CJ, Bui AQ, Chappie JS. 2020. The structure of the *Thermococcus gammatolerans* McrB N-terminal domain reveals a new mode of substrate recognition and specificity among McrB homologs. J Biol Chem 295:743–756. doi:10.1074/jbc.RA119.010188. 31822563 PMC6970917

[B14] Lacks S, Greenberg B. 1975. A deoxyribonuclease of *Diplococcus pneumoniae* specific for methylated DNA. J Biol Chem 250:4060–4066. doi:10.1016/S0021-9258(19)41386-0. 236309

[B15] Siwek W, Czapinska H, Bochtler M, Bujnicki JM, Skowronek K. 2012. Crystal structure and mechanism of action of the N6-methyladenine-dependent type IIM restriction endonuclease R.DpnI. Nucleic Acids Res 40:7563–7572. doi:10.1093/nar/gks428. 22610857 PMC3424567

[16] Mierzejewska K, Siwek W, Czapinska H, Kaus-Drobek M, Radlinska M, Skowronek K, Bujnicki JM, Dadlez M, Bochtler M. 2014. Structural basis of the methylation specificity of R.DpnI. Nucleic Acids Res 42:8745–8754. doi:10.1093/nar/gku546. 24966351 PMC4117772

[B17] Pingoud A, Wilson GG, Wende W. 2014. Type II restriction endonucleases—a historical perspective and more. Nucleic Acids Res 42:7489–7527. doi:10.1093/nar/gku447. 24878924 PMC4081073

[18] Xia YN, Burbank DE, Uher L, Rabussay D, Van Etten JL. 1986. Restriction endonuclease activity induced by PBCV-1 virus infection of a Chlorella-like green alga. Mol Cell Biol 6:1430–1439. doi:10.1128/mcb.6.5.1430-1439.1986. 3023890 PMC367667

[19] Xia Y, Burbank DE, Van Etten JL. 1986. Restriction endonuclease activity induced by NC-1A virus infection of a Chlorella-like green alga. Nucleic Acids Res 14:6017–6030. doi:10.1093/nar/14.15.6017. 3018667 PMC311618

[20] Zhang Y, Nelson M, Nietfeldt J, Xia Y, Burbank D, Ropp S, Van Etten JL. 1998. Chlorella virus NY-2A encodes at least 12 DNA endonuclease/methyltransferase genes. Virology 240:366–375. doi:10.1006/viro.1997.8936. 9454710

[21] Jeudy S, Rigou S, Alempic J-M, Claverie J-M, Abergel C, Legendre M. 2020. The DNA methylation landscape of giant viruses. Nat Commun 11:2657. doi:10.1038/s41467-020-16414-2. 32461636 PMC7253447

[22] Agarkova IV, Dunigan DD, Van Etten JL. 2006. Virion-associated restriction endonucleases of chloroviruses. J Virol 80:8114–8123. doi:10.1128/JVI.00486-06. 16873267 PMC1563800

[23] Atanasova NS, Heiniö CH, Demina TA, Bamford DH, Oksanen HM. 2018. The unexplored diversity of pleolipoviruses: the surprising case of two viruses with identical major structural modules. Genes (Basel) 9:131. doi:10.3390/genes9030131. 29495629 PMC5867852

[24] Demina TA, Oksanen HM. 2020. Pleomorphic archaeal viruses: the family *Pleolipoviridae* is expanding by seven new species. Arch Virol 165:2723–2731. doi:10.1007/s00705-020-04689-1. 32583077 PMC7547991

[25] Liu Y, Wang J, Liu Y, Wang Y, Zhang Z, Oksanen HM, Bamford DH, Chen X. 2015. Identification and characterization of SNJ2, the first temperate pleolipovirus integrating into the genome of the SNJ1-lysogenic archaeal strain. Mol Microbiol 98:1002–1020. doi:10.1111/mmi.13204. 26331239

[26] Demina TA, Atanasova NS, Pietilä MK, Oksanen HM, Bamford DH. 2016. Vesicle-like virion of *Haloarcula hispanica* pleomorphic virus 3 preserves high infectivity in saturated salt. Virology 499:40–51. doi:10.1016/j.virol.2016.09.002. 27632564

[27] Sencilo A, Paulin L, Kellner S, Helm M, Roine E. 2012. Related haloarchaeal pleomorphic viruses contain different genome types. Nucleic Acids Res 40:5523–5534. doi:10.1093/nar/gks215. 22396526 PMC3384331

[28] Sullivan MJ, Petty NK, Beatson SA. 2011. Easyfig: a genome comparison visualizer. Bioinformatics 27:1009–1010. doi:10.1093/bioinformatics/btr039. 21278367 PMC3065679

[29] Murray IA, Morgan RD, Luyten Y, Fomenkov A, Corrêa IR, Dai N, Allaw MB, Zhang X, Cheng X, Roberts RJ. 2018. The non-specific adenine DNA methyltransferase M.EcoGII. Nucleic Acids Res 46:840–848. doi:10.1093/nar/gkx1191. 29228259 PMC5778455

[30] Rousset F, Depardieu F, Miele S, Dowding J, Laval A-L, Lieberman E, Garry D, Rocha EPC, Bernheim A, Bikard D. 2022. Phages and their satellites encode hotspots of antiviral systems. Cell Host Microbe 30:740–753.e745. doi:10.1016/j.chom.2022.02.018. 35316646 PMC9122126

[31] Pausch P, Al-Shayeb B, Bisom-Rapp E, Tsuchida CA, Li Z, Cress BF, Knott GJ, Jacobsen SE, Banfield JF, Doudna JA. 2020. CRISPR-CasΦ from huge phages is a hypercompact genome editor. Science 369:333–337. doi:10.1126/science.abb1400. 32675376 PMC8207990

[32] Medvedeva S, Liu Y, Koonin EV, Severinov K, Prangishvili D, Krupovic M. 2019. Virus-borne mini-CRISPR arrays are involved in interviral conflicts. Nat Commun 10:5204. doi:10.1038/s41467-019-13205-2. 31729390 PMC6858448

[33] Luo G-Z, Wang F, Weng X, Chen K, Hao Z, Yu M, Deng X, Liu J, He C. 2016. Characterization of eukaryotic DNA N(6)-methyladenine by a highly sensitive restriction enzyme-assisted sequencing. Nat Commun 7:11301. doi:10.1038/ncomms11301. 27079427 PMC4835550

[34] Baek M, DiMaio F, Anishchenko I, Dauparas J, Ovchinnikov S, Lee GR, Wang J, Cong Q, Kinch LN, Schaeffer RD, Millán C, Park H, Adams C, Glassman CR, DeGiovanni A, Pereira JH, Rodrigues AV, van Dijk AA, Ebrecht AC, Opperman DJ, Sagmeister T, Buhlheller C, Pavkov-Keller T, Rathinaswamy MK, Dalwadi U, Yip CK, Burke JE, Garcia KC, Grishin NV, Adams PD, Read RJ, Baker D. 2021. Accurate prediction of protein structures and interactions using a three-track neural network. Science 373:871–876. doi:10.1126/science.abj8754. 34282049 PMC7612213

[35] Crooks GE, Hon G, Chandonia JM, Brenner SE. 2004. WebLogo: a sequence logo generator. Genome Res 14:1188–1190. doi:10.1101/gr.849004. 15173120 PMC419797

