# Supplementary material for: A Unique m6A-Dependent Restriction Endonuclease from an Archaeal Virus
Source: Microbiol Spectr. 2023 Mar 22;11(2):e04262-22. doi: 10.1128/spectrum.04262-22 (PMC10101028; doi:10.1128/spectrum.04262-22)
Supplement: Supplemental file 1 — Supplemental material. Download spectrum.04262-22-s0001.pdf, PDF file, 5.7 MB [file spectrum.04262-22-s0001.pdf]

Supplementary Materials

A unique m6A-dependent restriction endonuclease from an archaeal virus

Xueling Lu[1,#], Fengtao Huang[1,2,#,*], Rui Cheng[1], and Bin Zhu[1,2,*]

[1]Key Laboratory of Molecular Biophysics, the Ministry of Education, College of Life Science and Technology, Huazhong University of Science and Technology, Wuhan, Hubei 430074, China

[2]Shenzhen Huazhong University of Science and Technology Research Institute, Shenzhen 518063, China

[#]These authors contribute equally to this work.

*To whom correspondence should be addressed. Email: huang_fengtao@126.com; bin_zhu@hust.edu.cn

Table S1. The primers for amplification of the DNA fragments from pBR322 plasmid, T7 phage genome or λ DNA.

Name	Primers (F/R 5′-3′)	Template	Size
F1	GCAGGCCATGCTGTCCAGGCAGGTAGATGACGACCATCAG<br>AGTTGGGTGCACGAGTGGGTTACATCGAACTGGATCTCAA	pBR322 plasmid	3 kb
F2	AGAAGCAGGCCATTATCGCCGGC<br>CGGCGCCTACAATCCATGCCAACCC	pBR322 plasmid	300 bp
F3	CGTGCTCCTGTCGTTGAGGACCCGGCTAGG<br>CGGTAAAGCTCATCAGCGTGGTCG	pBR322 plasmid	600 bp
F4	CTCCACGCGGTGCAATCGTTGCCGATAAGACCAACATG<br>CCCCGCAGTGGAACTTAGTGACGCTCTCTAAGAGGG	T7 phage genome	177 bp
λ DNA-1	GGGCGGCGACCTCGCGGGTTTTCGCTATTTATG<br>CGCGACAGCACGAAAGTACAGAATGCGGTTTC	λ DNA	549 bp
λ DNA-2	CCGCATTTTATGCGTTTTCATGTTGCCTGCCCG<br>CAGCTTTCCTCACCCGGCCCCCATCCCCATACGC	λ DNA	572 bp
λ DNA-3	ACGGGAGGCGCTGTGGCTGATTTCGATAACC<br>TGGCCCTTTTCAGCCTGGCCCTTTCCTTTACCAG	λ DNA	570 bp
λ DNA-4	GATAGTGCGGGTGTTGAATGATTTCCAGTTGC<br>ACTGGAGGCAGGAAGACAAACACAGAGCTC	λ DNA	589 bp
λ DNA-5	AAGCCATGAATGTAACGTAACGGAATTATCAC<br>TGCAGACGTAACCAATATTCGAATTGAAGAAC	λ DNA	530 bp
λ DNA-6	CCAACAAGCCGTAAACGCCTTCATCAGAG<br>GCCATCAATTTTTTCGTAATAGCGCATCTC	λ DNA	568 bp
λ DNA-7	TACCTACAAAGCCCAGCGCGACAAAAATGCC<br>CTTCGTTTCTGGAATTGGGCAGAAGAAAAC	λ DNA	573 bp
T7 DNA-8	TCAAGCGAGACGGTACTGTGGAGGCAGGAC<br>CACCGTCTACTTTGGCAATCCAGTAGCCAG	T7 phage genome	1018 bp
T7 DNA-9	TTCGCAACGGTAAGGCGACTATGGTTTACCGCTG<br>ATCTCGCCTAAGCGATAACCCCACGCCTCCAAAGC	T7 phage genome	1100 bp
T7 DNA-10	CTGAGACTTTCAGAAACCAAGCGGAGGGC<br>CTGTGAAACAGTCACACTTACCCCACCGCC	T7 phage genome	941 bp
T7-3 kb	CTCTTTCGTTACGTGAACGAATCCGTGAGCACCTA<br>TTAAACACAACATGTTCAACTGGGGTGTAAGGAG	T7 phage genome	3 kb
T7-2 kb	GTGGTATCGGCTCTTTCGTTACGTGAACGAATCCG<br>TCATATTGATTTCTCCTATTGATTATCGTGAC	T7 phage genome	2 kb
T7-1 kb	TCGTTTCTGACATCGAAGCTAAC<br>TTAAACACAACATGTTCAACTGGGGTGTAAGGAG	T7 phage genome	1 kb

pBR-1.2 kb	AGTCCAACCCGGTAAGACACGAC<br>AGTTGGGTGCACGAGTGGGTTACATCGAAC	pBR322 plasmid	1.2 kb

Figure S1. SDS-PAGE analysis of the purified HHPV4I and its mutants. Related to Figure 3.

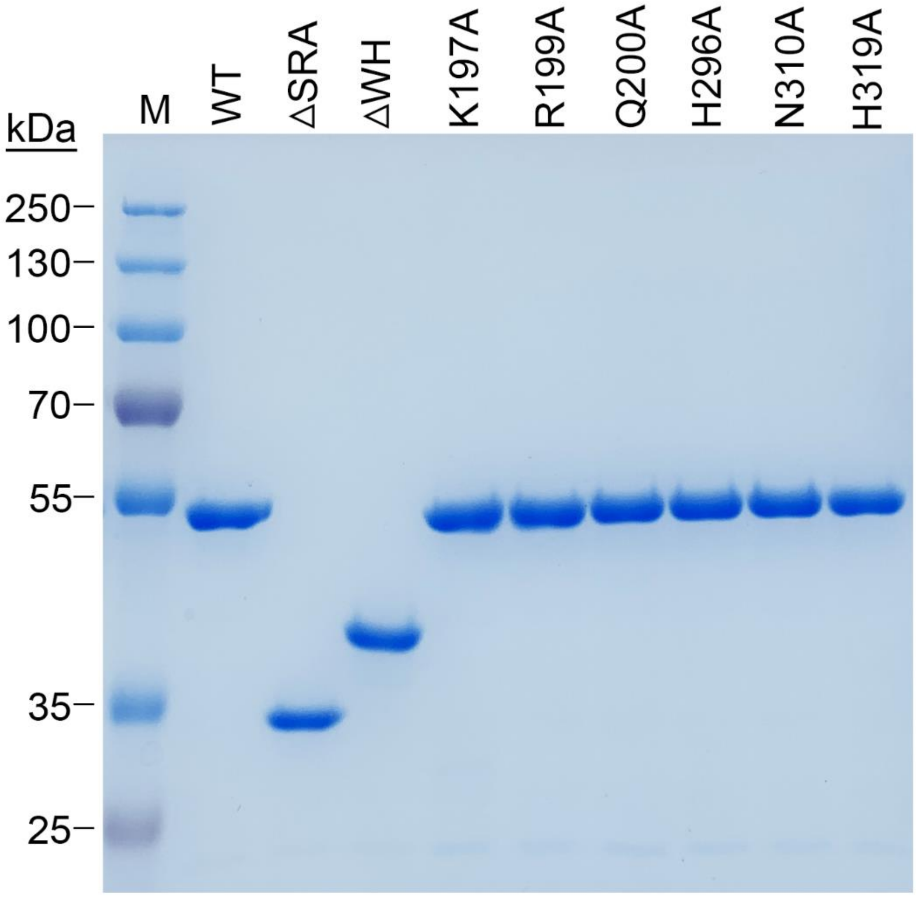

Figure S2. Determination of the△SRA-generated cleavage sites by run-off sequencing. (A) Agarose gel showing that the Dam-methylated DNA F3 digested by △SRA for 15 min, 30 min and 60 min. (B–D) The Dam-methylated DNA F3 was digested by HHPV4I for 15 min (B), 30 min (C) and 60 min (D), and then the generated cleavage sites were determined by using run-off sequencing.

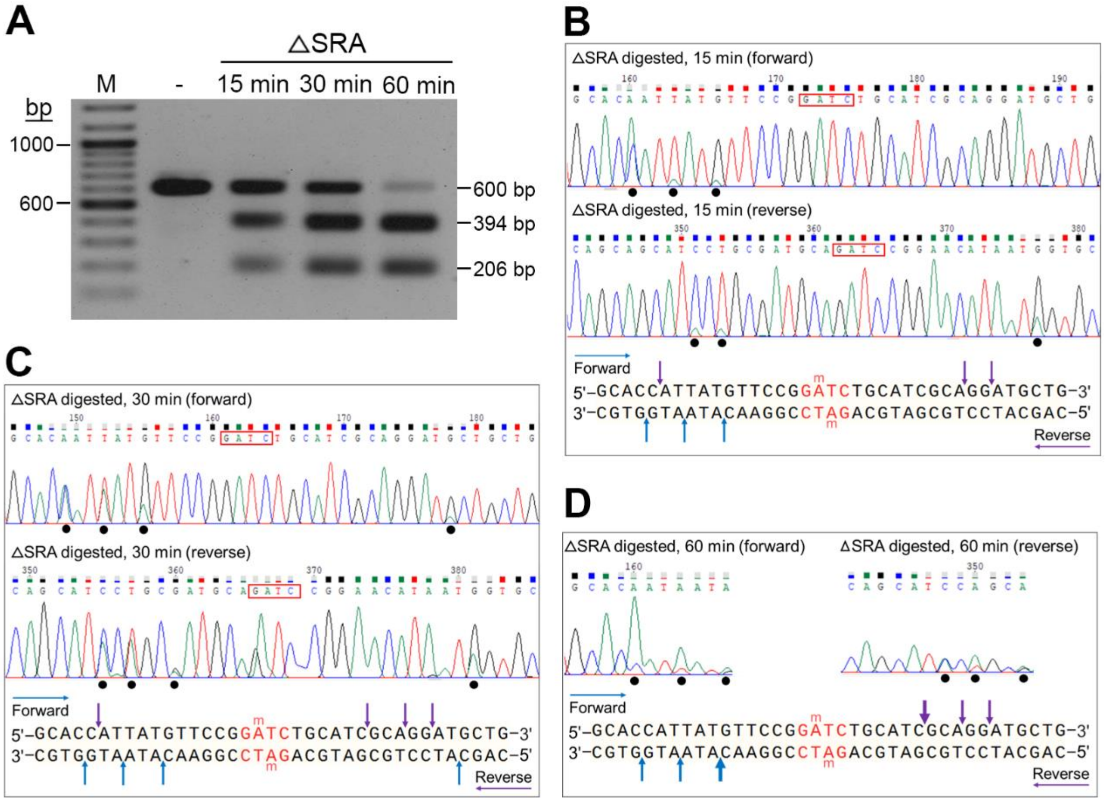

Figure S3. Determination of the cleavage sites generated by HHPV4I digestion for 15 min. (A) Different Dam-methylated DNA fragments were subjected to HHPV4I digestion for 15 min. After digestion, the DNA fragments were purified by using Qiaquick PCR purification kit (Qiagen), and then analyzed by agarose gel electrophoresis. (B) The purified DNA fragments were subjected to run-off sequencing.

A

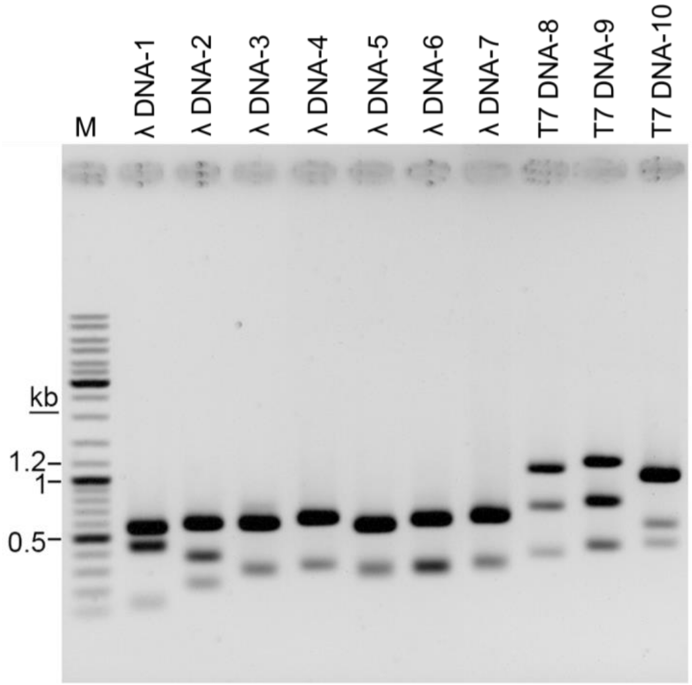

B

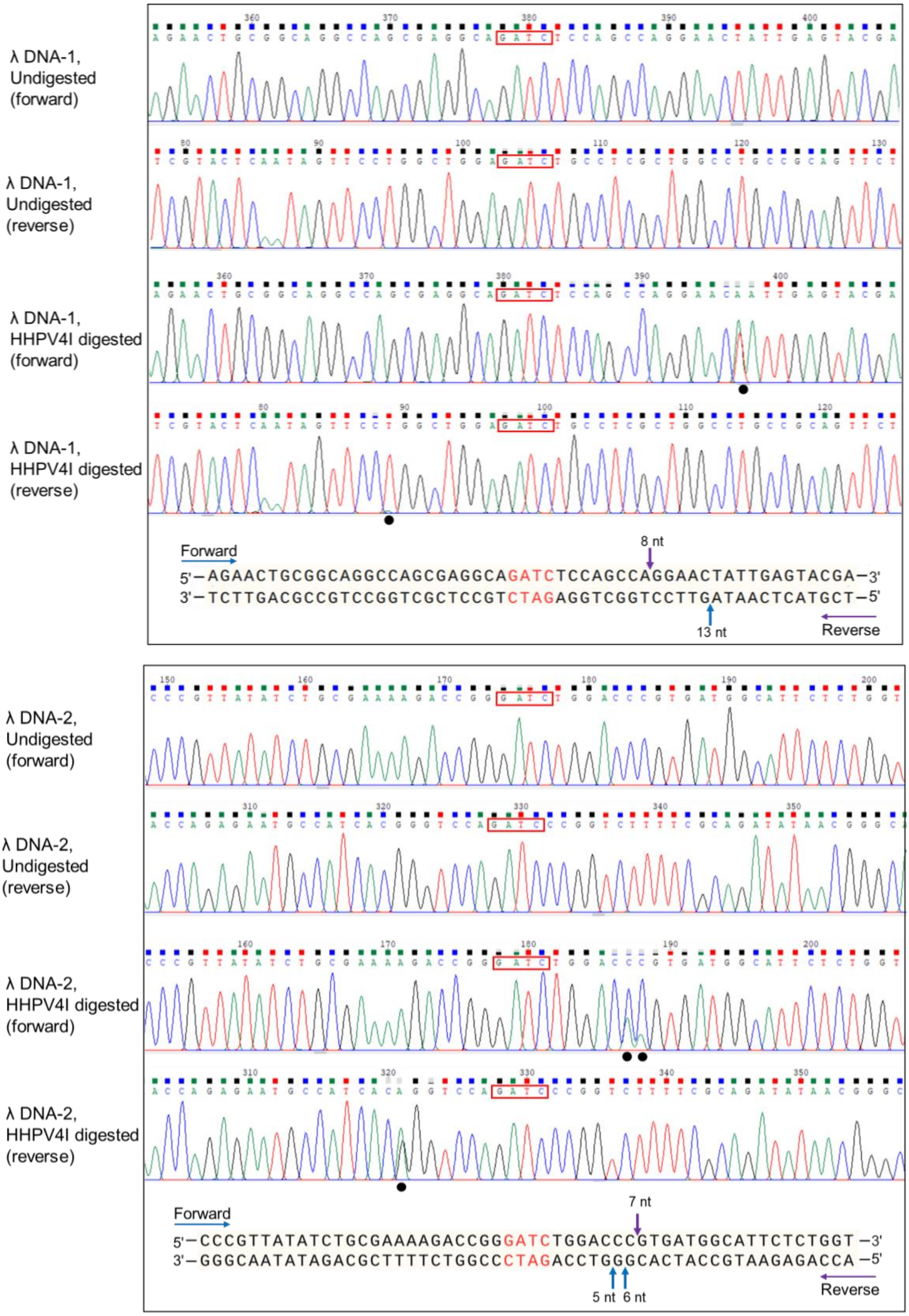
λ DNA-1, Undigested (forward)
λ DNA-1, Undigested (reverse)
λ DNA-1, HHPV4I digested (forward)
λ DNA-1, HHPV4I digested (reverse)
Forward
8 nt
5'–AGAACTGCGGCAGGCCAGCGAGGCAGATCTCCAGCCAGGAACTATTGAGTACGA–3'
3'–TCTTGACGCCGTCCGGTCGCTCCGTCTAGAGGTCGGTCCTTGATAACTCATGCT–5'
13 nt
Reverse
λ DNA-2, Undigested (forward)
λ DNA-2, Undigested (reverse)
λ DNA-2, HHPV4I digested (forward)
λ DNA-2, HHPV4I digested (reverse)
Forward
7 nt
5'–CCCGTTATATCTGCGAAAAGACCGGGATCTGGACCCGTGATGGCATTCTCTGGT–3'
3'–GGGCAATATAGACGCTTTTCTGGCCCTAGACCTGGGCACTACCGTAAGAGACCA–5'
5 nt 6 nt
Reverse

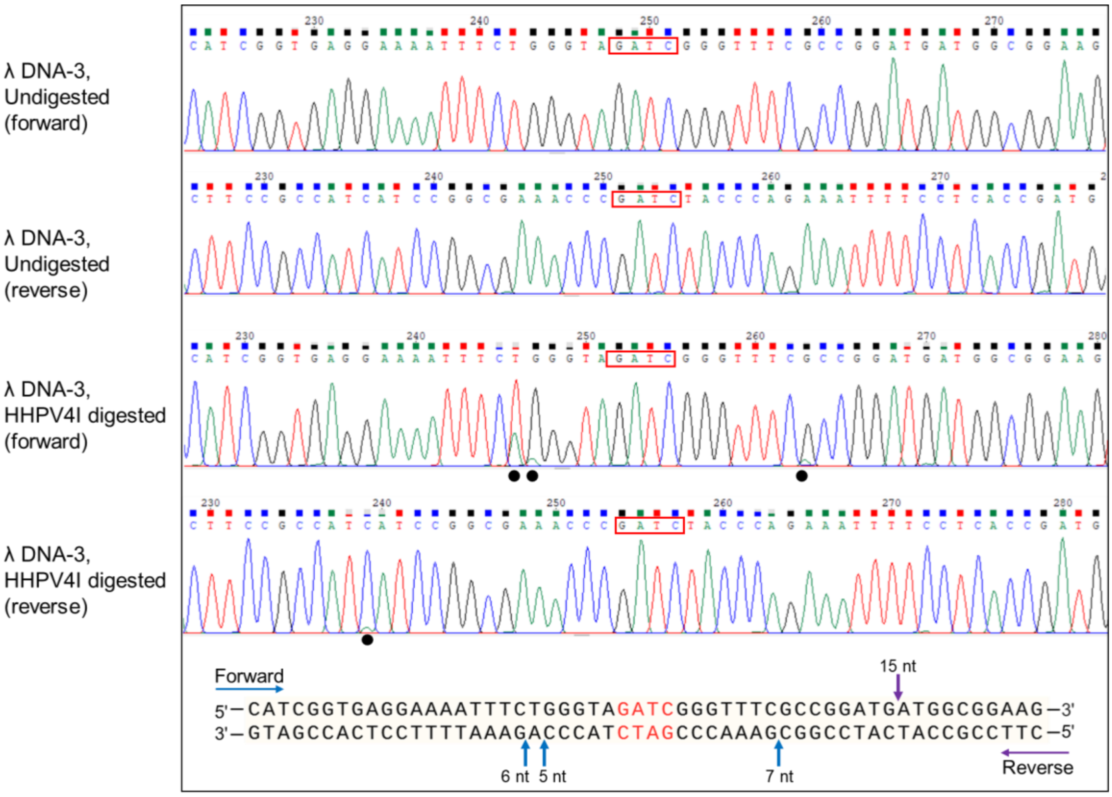
λ DNA-3, Undigested (forward)
λ DNA-3, Undigested (reverse)
λ DNA-3, HHPV4I digested (forward)
λ DNA-3, HHPV4I digested (reverse)
Forward
15 nt
5'—CATCGGTGAGGAAAATTTCTGGGTAGATCGGGTTTCGCCGGATGATGGCGGAAG—3'
3'—GTAGCCACTCCTTTTAAAGACCCATCTAGCCCAAAGCGGCCTACTACCGCCTTC—5'
6 nt
5 nt
7 nt
Reverse

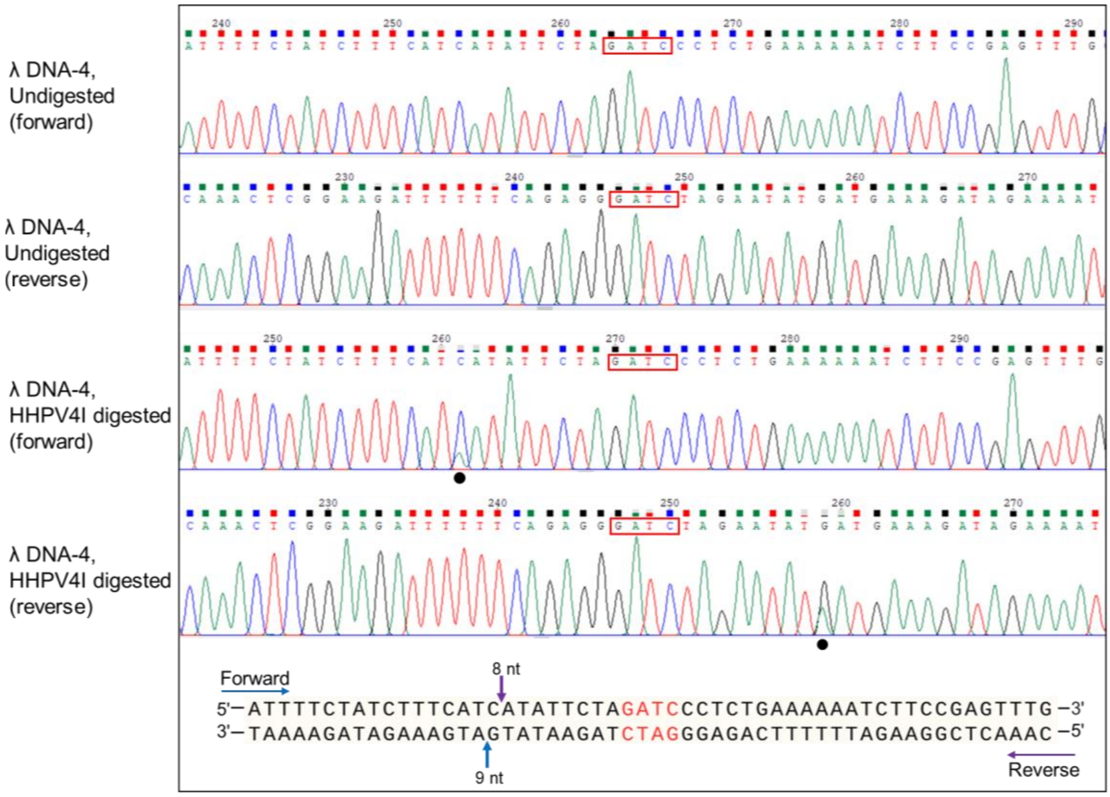
λ DNA-4, Undigested (forward)
λ DNA-4, Undigested (reverse)
λ DNA-4, HHPV4I digested (forward)
λ DNA-4, HHPV4I digested (reverse)
Forward
8 nt
5'—ATTTTCTATCTTTCATCATATTCTAGATCCCTCTGAAAAAATCTTCCGAGTTTG—3'
3'—TAAAAGATAGAAAGTAGTATAAGATCTAGGGAGACTTTTTTAGAAGGCTCAAAC—5'
9 nt
Reverse

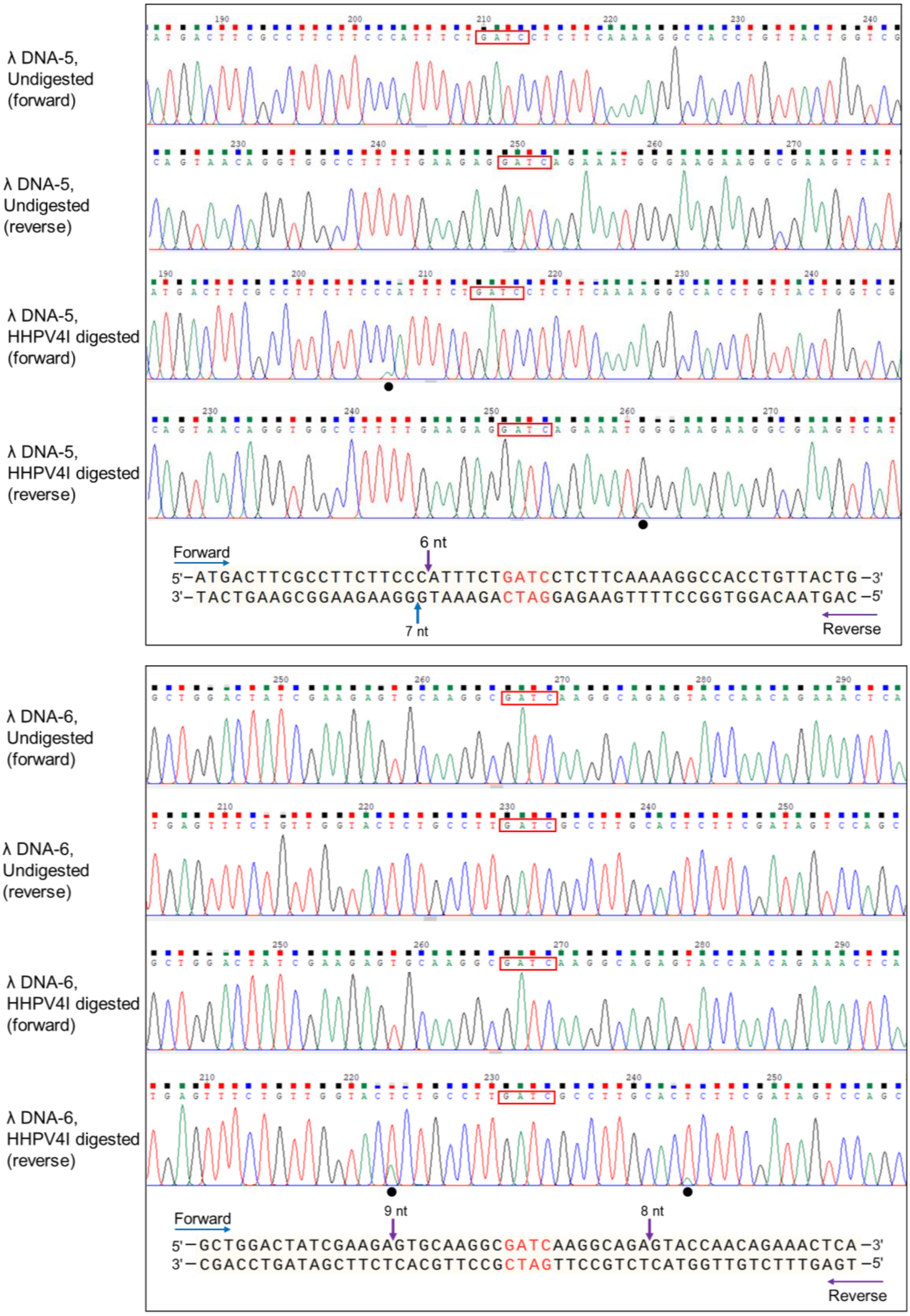

λ DNA-5, Undigested (forward)
λ DNA-5, Undigested (reverse)
λ DNA-5, HHPV4I digested (forward)
λ DNA-5, HHPV4I digested (reverse)
Forward
6 nt
5′–ATGACTTCGCCTTCTTCCCATTTCTGATCCTCTTCAAAAGGCCACCTGTTACTG–3′
3′–TACTGAAGCGGAAGAAGGGTAAAGACTAGGAGAAGTTTTCCGGTGGACAATGAC–5′
7 nt
Reverse
λ DNA-6, Undigested (forward)
λ DNA-6, Undigested (reverse)
λ DNA-6, HHPV4I digested (forward)
λ DNA-6, HHPV4I digested (reverse)
Forward
9 nt
8 nt
5′–GCTGGACTATCGAAGAGTGCAAGGCGATCAAGGCAGAGTACCAACAGAAACTCA–3′
3′–CGACCTGATAGCTTCTCACGTTCCGCTAGTTCCGTCTCATGGTTGTCTTTGAGT–5′
Reverse

λ DNA-7, Undigested (forward)

λ DNA-7, Undigested (reverse)

λ DNA-7, HHPV4I digested (forward)

λ DNA-7, HHPV4I digested (reverse)

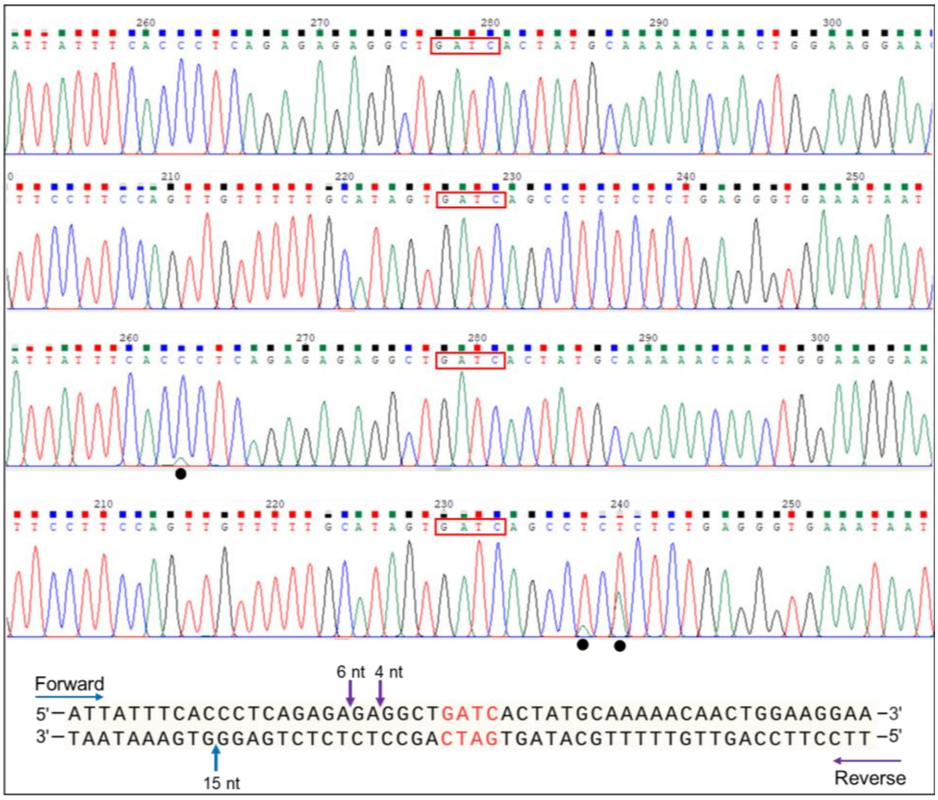

T7 DNA-8, Undigested (forward)

T7 DNA-8, Undigested (reverse)

T7 DNA-8, HHPV4I digested (forward)

T7 DNA-8, HHPV4I digested (reverse)

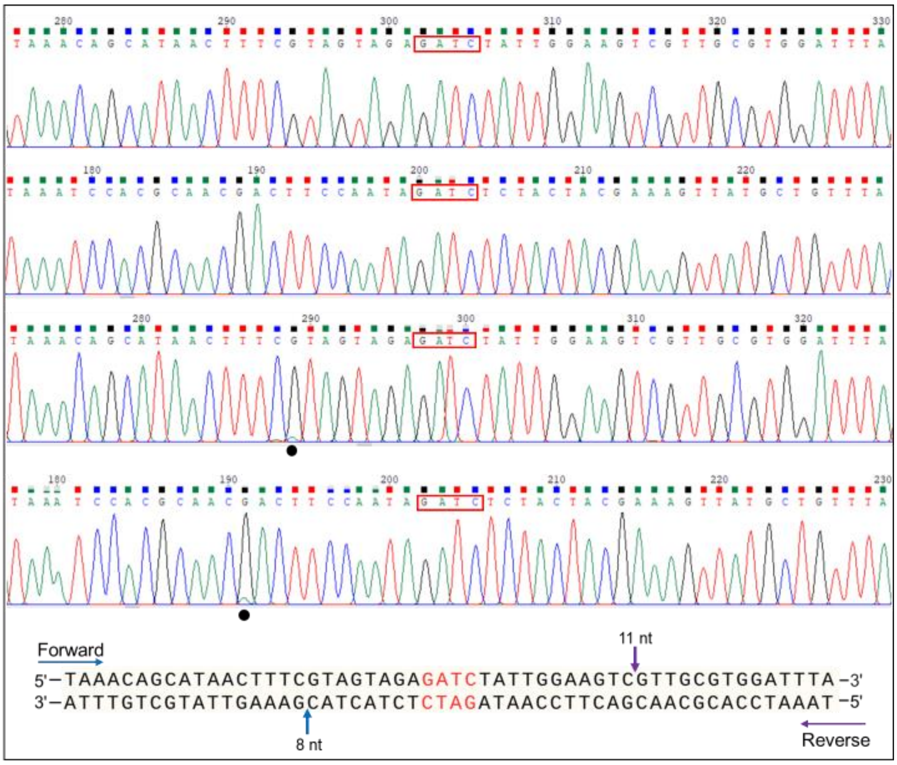

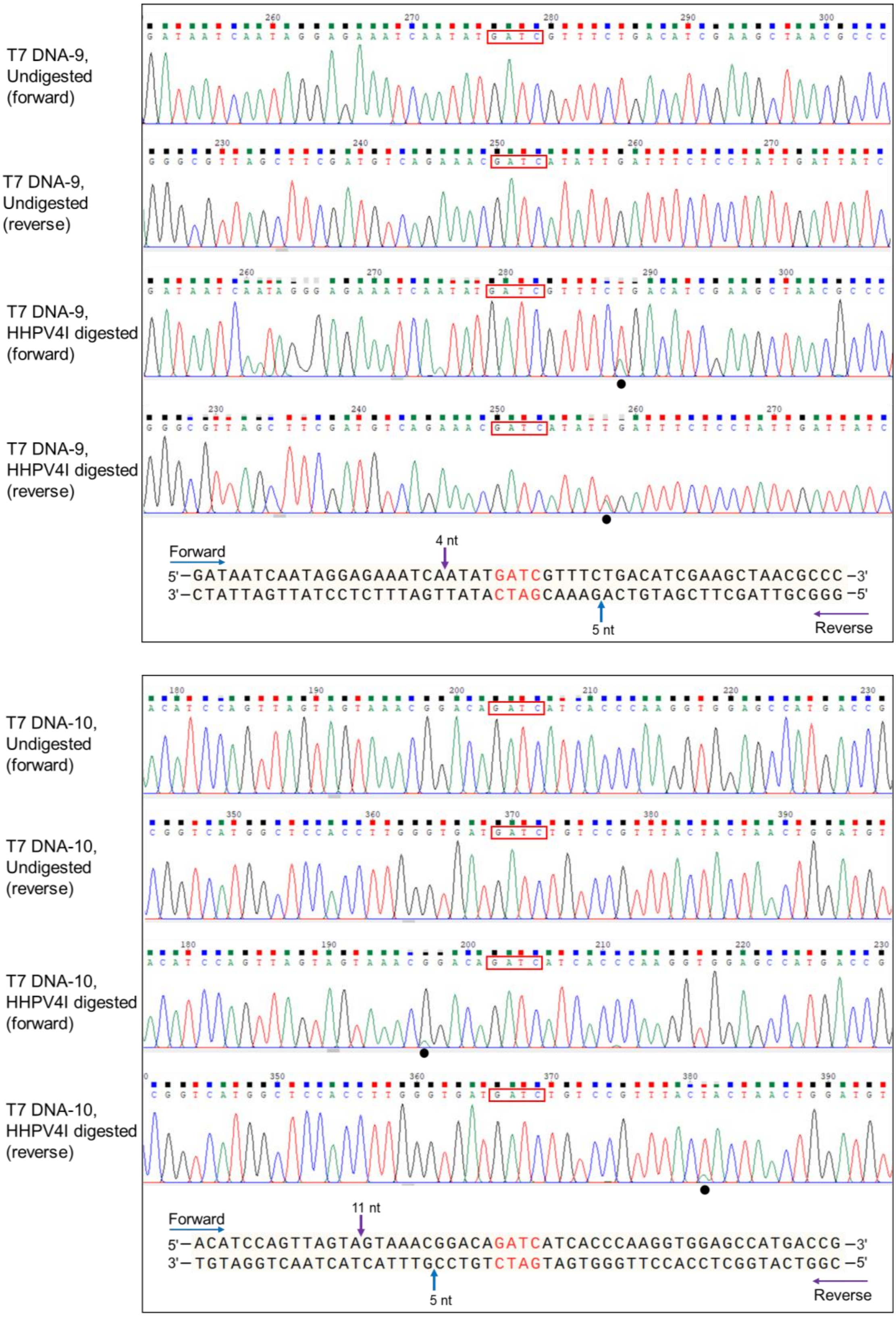
T7 DNA-9, Undigested (forward)
T7 DNA-9, Undigested (reverse)
T7 DNA-9, HHPV4I digested (forward)
T7 DNA-9, HHPV4I digested (reverse)
Forward
4 nt
5'–GATAATCAATAGGAGAAATCAATATGATCGTTTCTGACATCGAAGCTAACGCCC–3'
3'–CTATTAGTTATCCTCTTTAGTTATACTAGCAAAGACTGTAGCTTCGATTGCGGG–5'
5 nt
Reverse
T7 DNA-10, Undigested (forward)
T7 DNA-10, Undigested (reverse)
T7 DNA-10, HHPV4I digested (forward)
T7 DNA-10, HHPV4I digested (reverse)
Forward
11 nt
5'–ACATCCAGTTAGTAGTAAACGGACAGATCATCACCCAAGGTGGAGCCATGACCG–3'
3'–TGTAGGTCAATCATCATTTGCCTGTCTAGTAGTGGGTTCCACCTCGGTACTGGC–5'
5 nt
Reverse

Figure S4. Determination of the cleavage sites generated by HHPV4I digestion for 30 min. (A) Different Dam-methylated DNA fragments were digested by HHPV4I for 30 min. After digestion, the DNA fragments were purified by using Qiaquick PCR purification kit (Qiagen), and then were analyzed by agarose gel electrophoresis. (B) The purified DNA fragments were subjected to run-off sequencing.

A

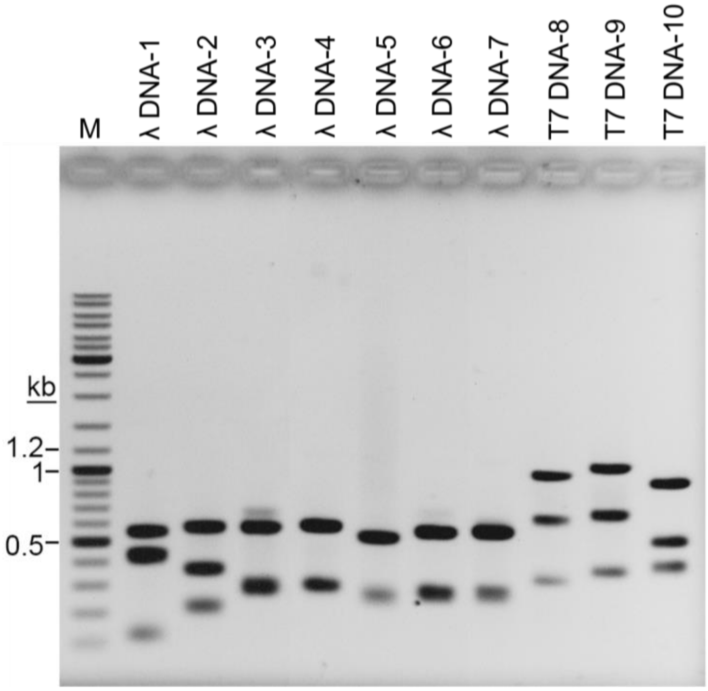

B

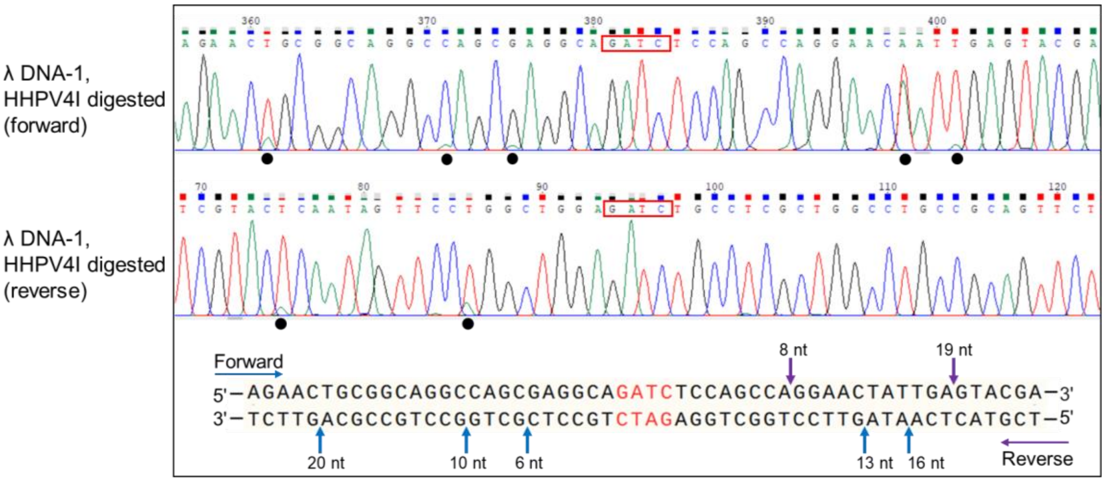

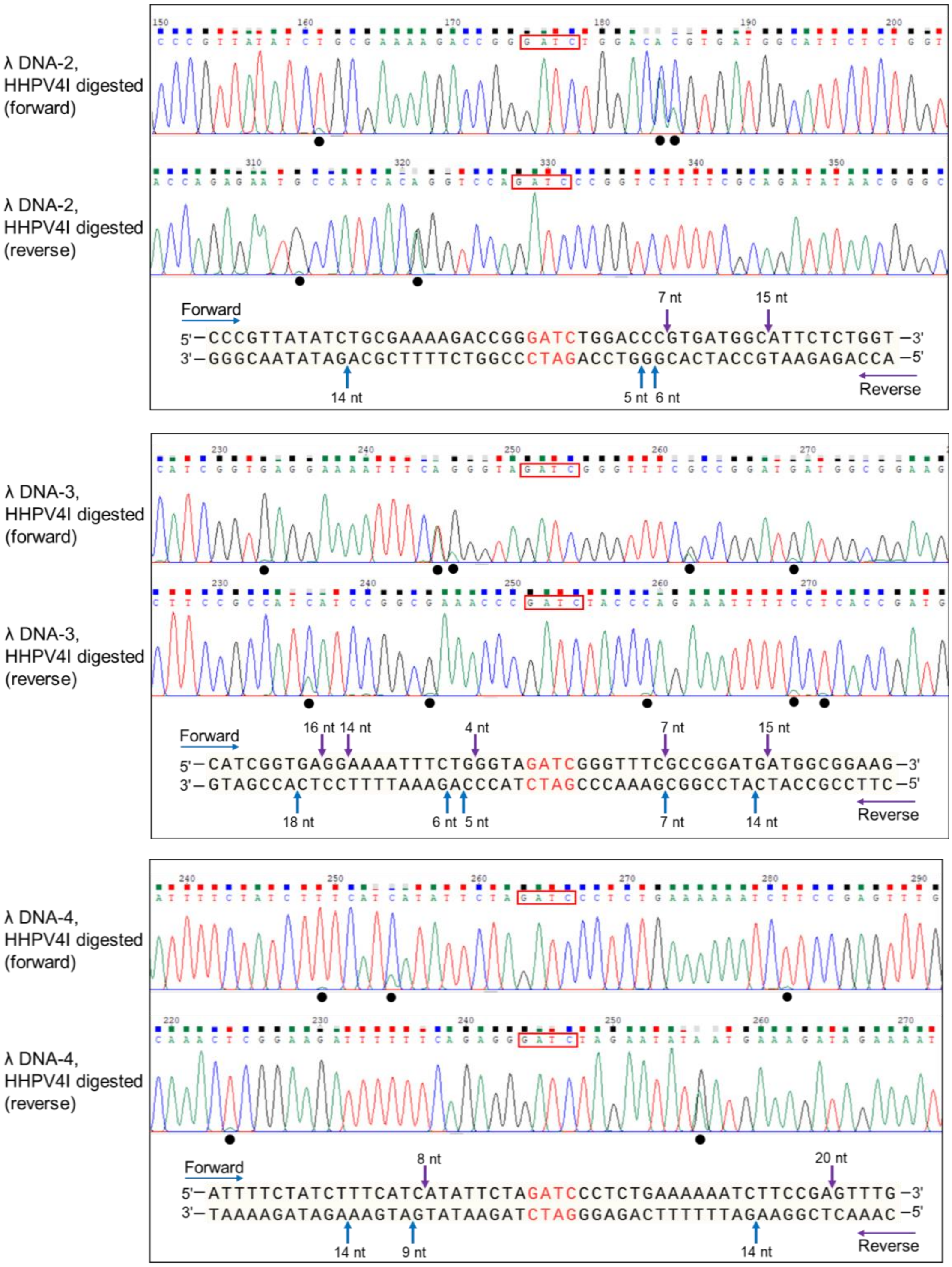
λ DNA-2, HHPV4I digested (forward)
λ DNA-2, HHPV4I digested (reverse)
Forward
7 nt
15 nt
5'–CCCGTTATATCTGCGAAAAGACCGGGATCTGGACCCGTGATGGCATTCTCTGGT–3'
3'–GGGCAATATAGACGCTTTTCTGGCCCTAGACCTGGGCACTACCGTAAGAGACCA–5'
14 nt
5 nt
6 nt
Reverse
λ DNA-3, HHPV4I digested (forward)
λ DNA-3, HHPV4I digested (reverse)
Forward
16 nt
14 nt
4 nt
7 nt
15 nt
5'–CATCGGTGAGGAAAATTTCTGGGTAGATCGGGTTTCGCCGGATGATGGCGGAAG–3'
3'–GTAGCCACTCCTTTTAAAGACCCATCTAGCCCAAAGCGGCCTACTACCGCCTTC–5'
18 nt
6 nt
5 nt
7 nt
14 nt
Reverse
λ DNA-4, HHPV4I digested (forward)
λ DNA-4, HHPV4I digested (reverse)
Forward
8 nt
20 nt
5'–ATTTTCTATCTTTCATCATATTCTAGATCCCTCTGAAAAAATCTTCCGAGTTTG–3'
3'–TAAAAGATAGAAAGTAGTATAAGATCTAGGGAGACTTTTTTAGAAGGCTCAAAC–5'
14 nt
9 nt
14 nt
Reverse

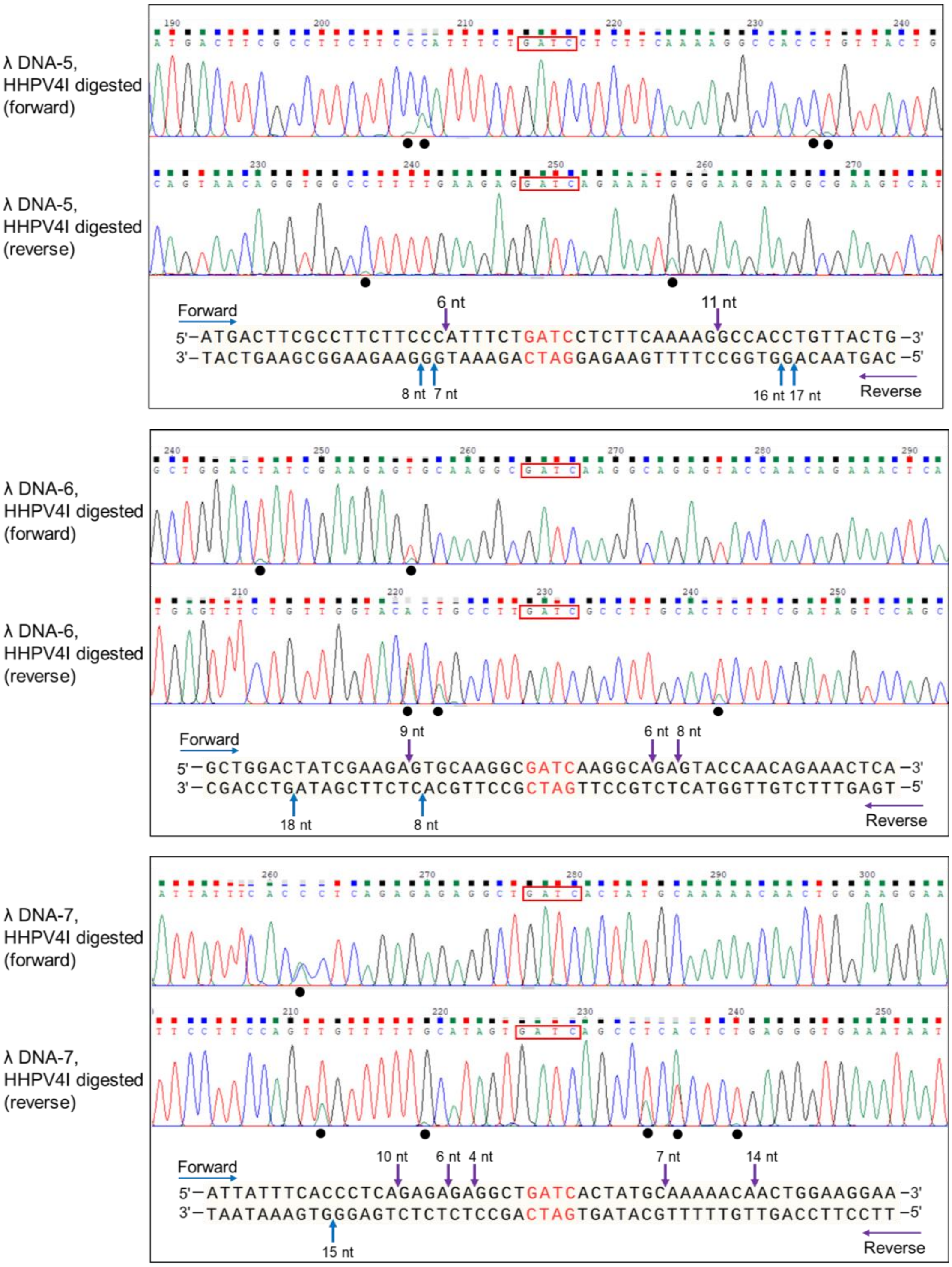
λ DNA-5, HHPV4I digested (forward)
λ DNA-5, HHPV4I digested (reverse)
Forward
6 nt
11 nt
5'-ATGACTTCGCCTTCTTCCCATTTCTGATCCTCTTCAAAAGGCCACCTGTTACTG-3'
3'-TACTGAAGCGGAAGAAGGGTAAAGACTAGGAGAAGTTTTCCGGTGGACAATGAC-5'
8 nt 7 nt
16 nt 17 nt
Reverse
λ DNA-6, HHPV4I digested (forward)
λ DNA-6, HHPV4I digested (reverse)
Forward
9 nt
6 nt 8 nt
5'-GCTGGACTATCGAAGAGTGCAAGGCGATCAAGGCAGAGTACCAACAGAAACTCA-3'
3'-CGACCTGATAGCTTCTCACGTTCCGCTAGTTCCGTCTCATGGTTGTCTTTGAGT-5'
18 nt
8 nt
Reverse
λ DNA-7, HHPV4I digested (forward)
λ DNA-7, HHPV4I digested (reverse)
Forward
10 nt
6 nt 4 nt
7 nt
14 nt
5'-ATTATTTCACCCTCAGAGAGAGGCTGATCACTATGCAAAAACAACTGGAAGGAA-3'
3'-TAATAAAGTGGGAGTCTCTCTCCGACTAGTGATACGTTTTTGTTGACCTTCCTT-5'
15 nt
Reverse

T7 DNA-8, HHPV4I digested (forward)

T7 DNA-8, HHPV4I digested (reverse)

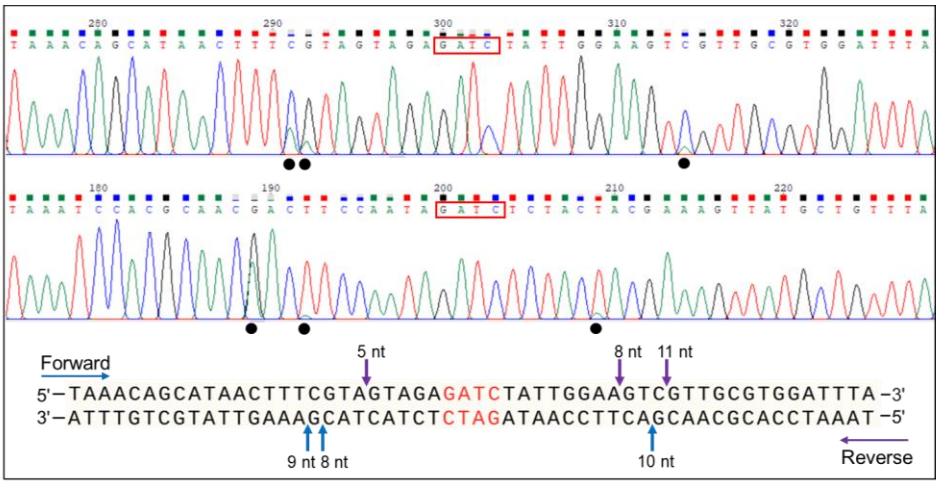

T7 DNA-9, HHPV4I digested (forward)

T7 DNA-9, HHPV4I digested (reverse)

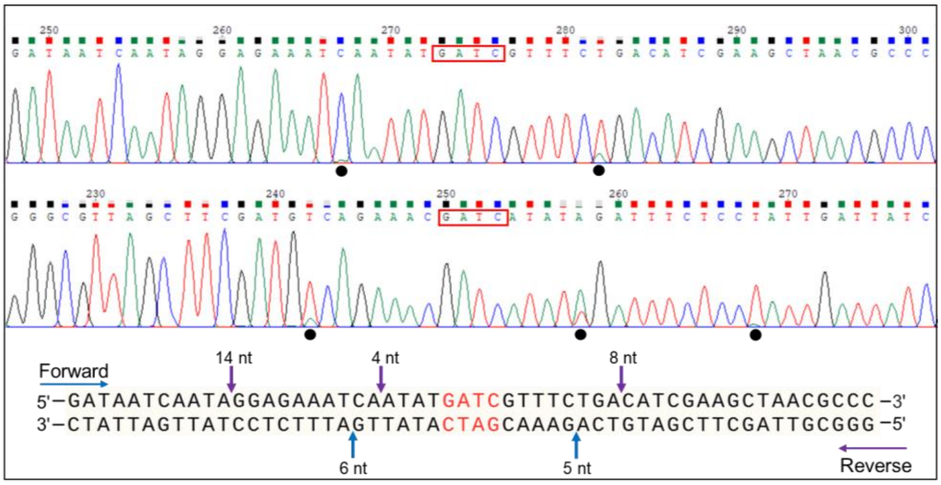

T7 DNA-10, HHPV4I digested (forward)

T7 DNA-10, HHPV4I digested (reverse)

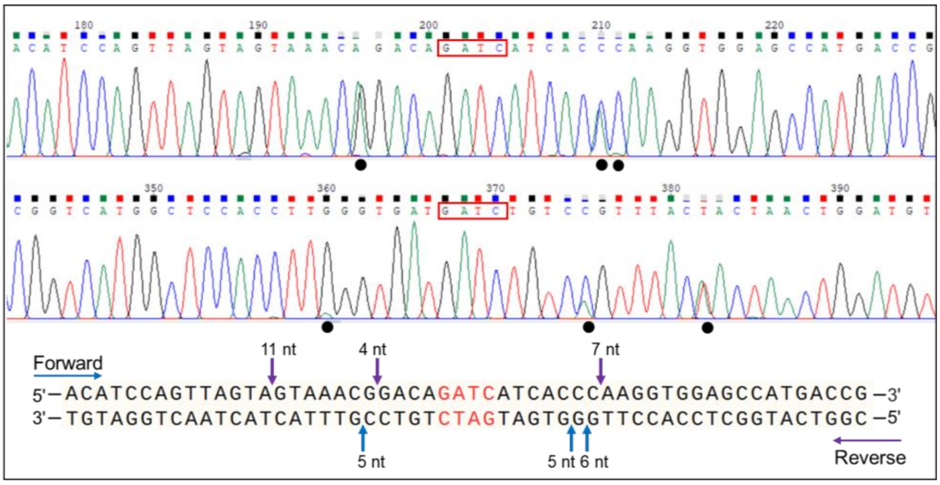

Figure S5. Determination of the cleavage sites generated by K197A digestion. (A) M. EcoGII-methylated T7-1 kb DNA was digested by K197A for 30 min. After digestion, the DNA fragments were purified by using Qiaquick PCR purification kit (Qiagen), and then the purified DNA fragments were subjected to run-off sequencing. Four primers (Forward primer 1, Forward primer 2, Reverse primer 1, and Reverse primer 2) are used for sequencing of the full-length of the two DNA strands. (B) Summary of the positions of the cleavage sites on the T7-1 kb DNA. Green arrows and red arrows indicate the positions of the cleavage sites on the forward DNA strand and reverse DNA strand, respectively. (C) Summary of the cleavage site sequences.

A

(Forward primer 1)

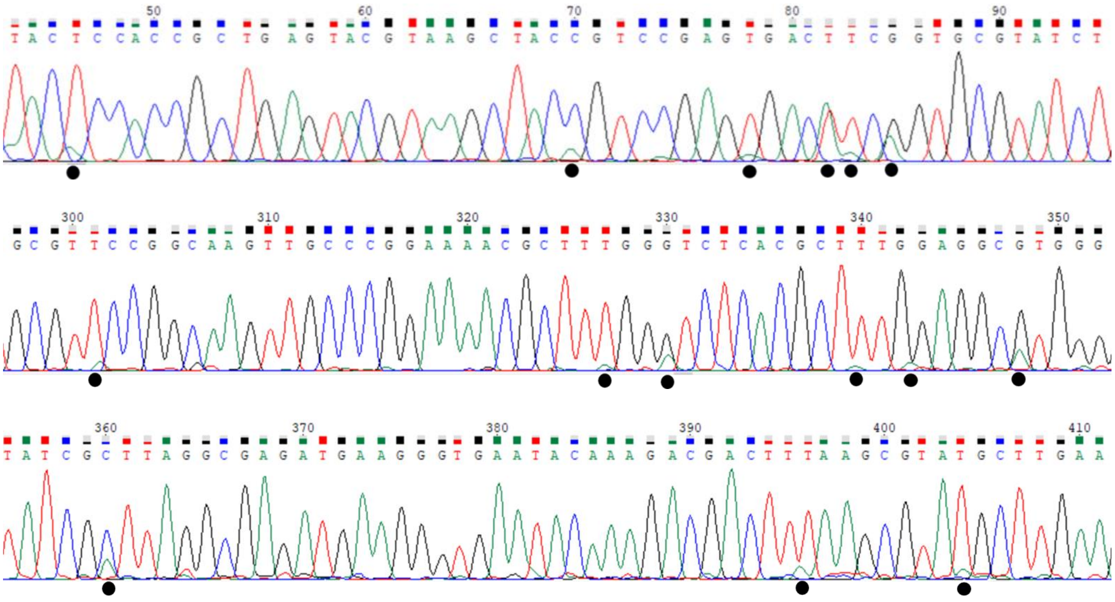

(Forward primer 2)

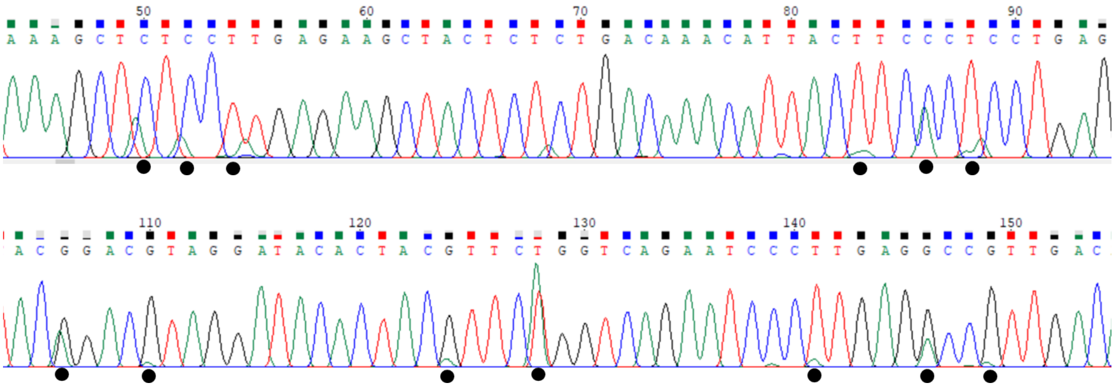

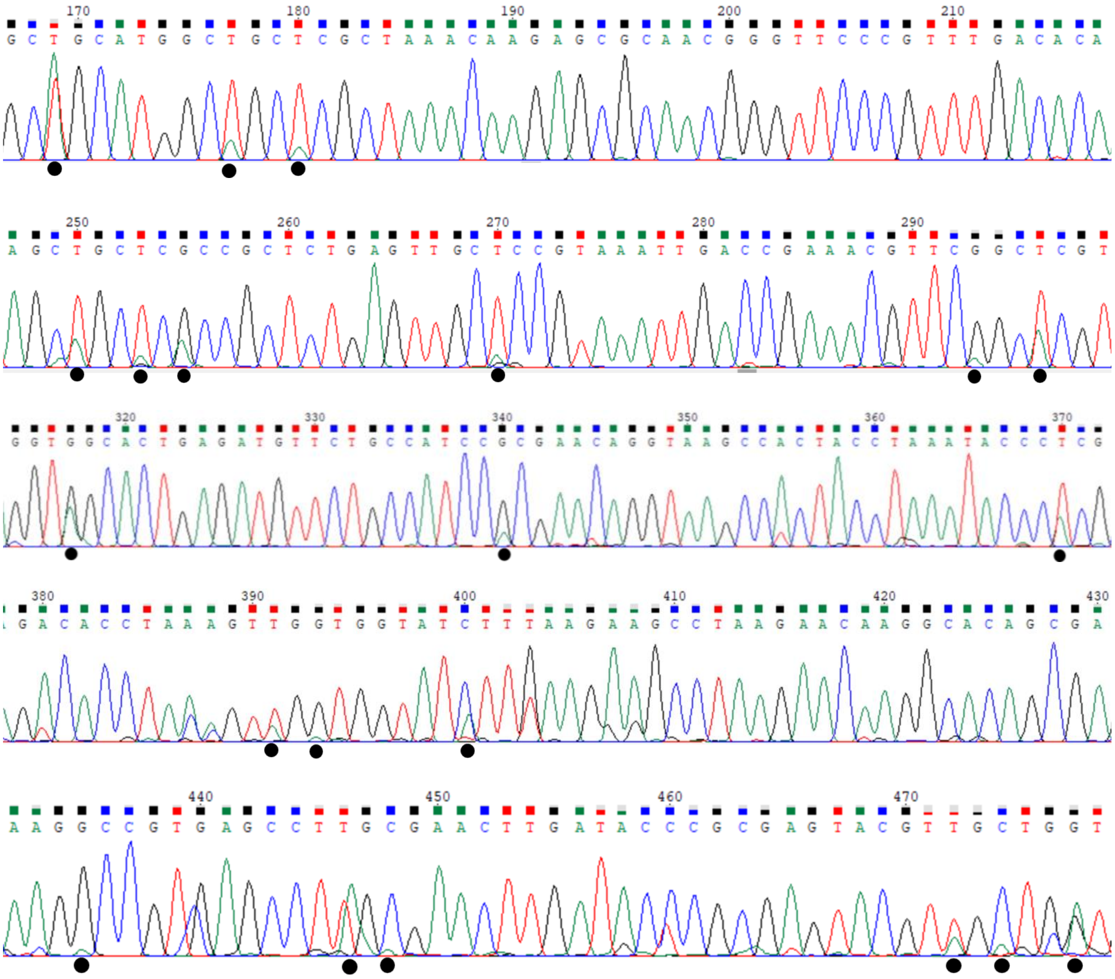

(Reverse primer 1)

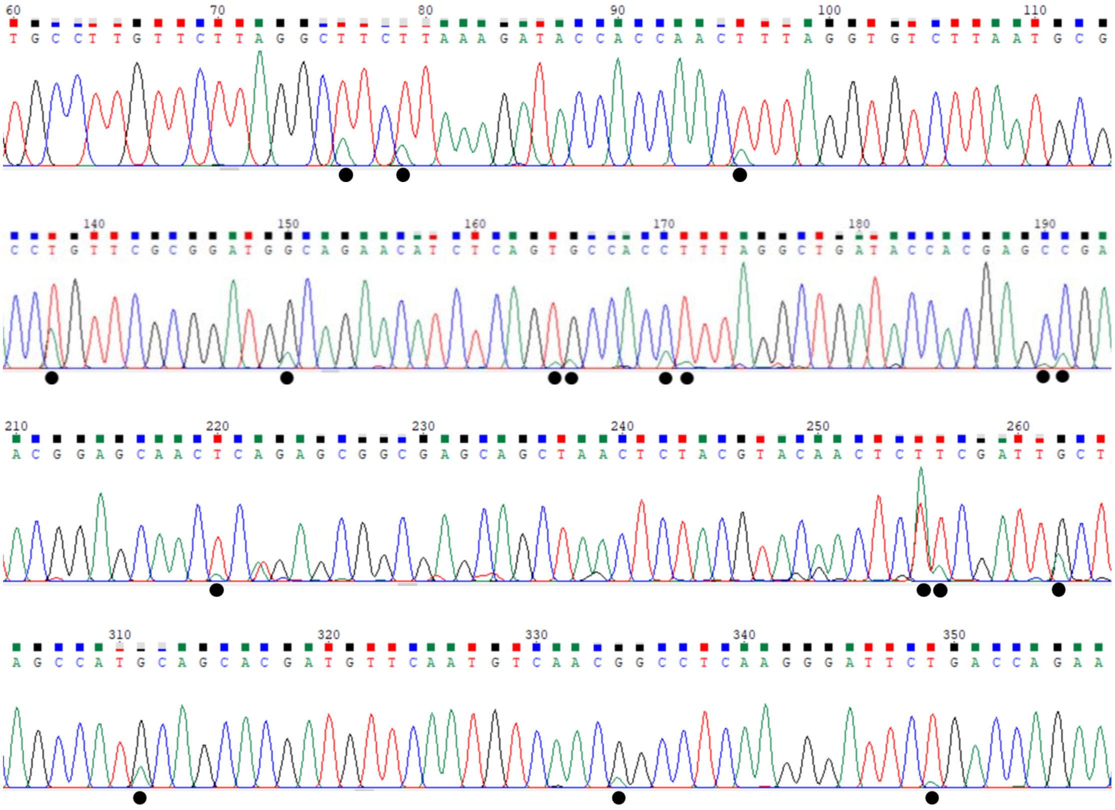

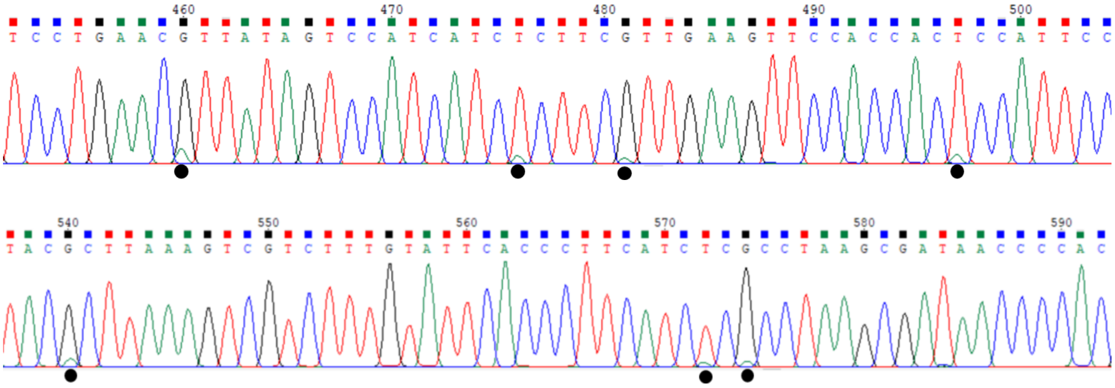

(Reverse primer 2)

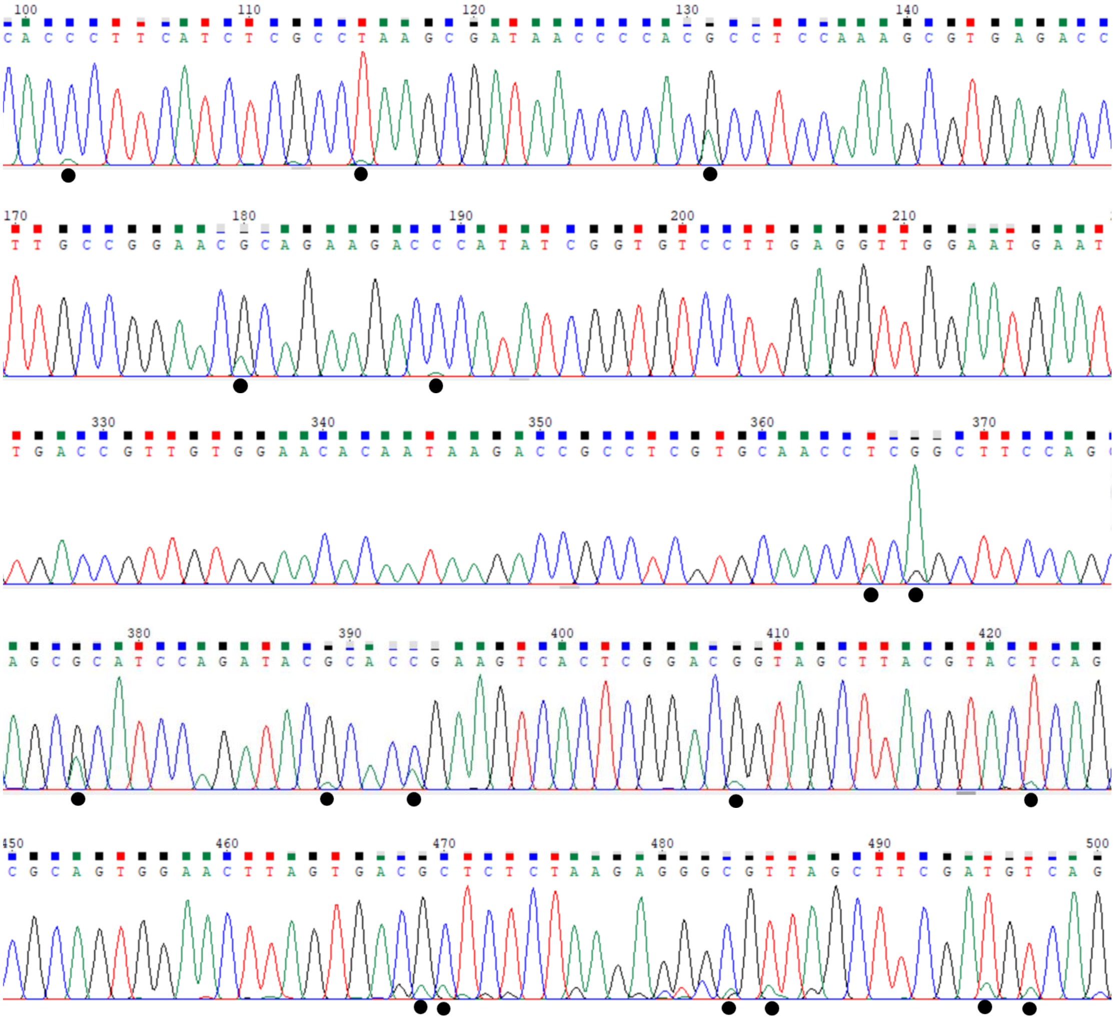

B

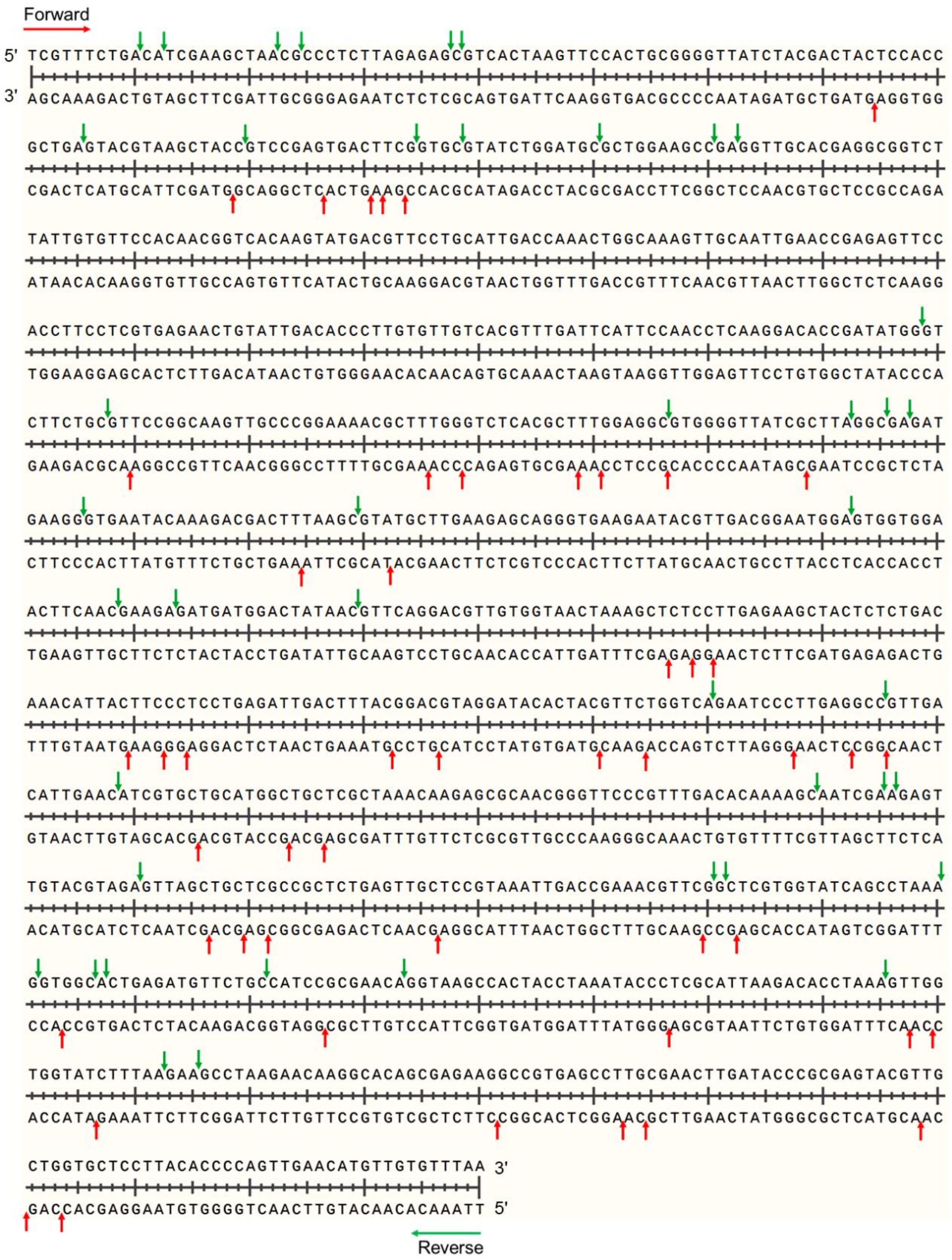

C

GGAGTA	GCATAC	AACGGC	CCAACT	TGGGTC	AACATC	GCCGTT
ACGGTA	GAGAGC	GCAGCA	AAGATA	GAGCGT	AGCAAT	CGAAGA
GAAGTC	AGGAGA	GCAGCC	CACCAA	AGCGTC	AGGGTG	GAAGAG
ACCGAA	AGGGAA	CGAGCA	GGCCTT	TGAGTA	GGCGAG	AGAGTT
TCACTC	GGAGGG	GCAGCT	GCAACG	ACCGTC	CGAGAT	TCGGCT
CGAAGT	CAAGGA	CGAGCA	CAGCAA	TCGGTG	AGCGTA	AAGGTG
GGAACG	GAAGTA	GGCGAG	CACCAG	TGCGTA	GGAGTG	GGCACT
CCAAAG	TCCGTA	GGAGCA	GCAAGG	TGCGCT	AACGAA	GCACTG
GACCCA	TACGTC	GCCGAA	TCGCAA	GCCGAG	AGAGAT	TGCCAT
CAAAGC	AACGTA	CGAGCC	TGACAT	CGAGGT	AACGTT	ACAGGT
TCCAAA	CCAGAA	GCCACC	ACATCG	TGCGTT	TCAGAA	AAAGTT
CACGCC	CAAGGG	CGCGGA	TAACGC	GGCGTG	AAAGGT	TAAGAA
AAGCGA	GGCCTC	CGAGGG	ACGCCC	TTAGGC	CGGCTC	GAAGCC
TTAAAG						

Figure S6. Examination of the HHPV4I cleavage activity towards DNA with different cytosine modifications. (A) cytosine, (B) 5mC or (C) 5hmC-containing 1.2 kb DNA fragments were amplified from pBR322 plasmid (pBR-1.2 kb, Table S3). The 5hmC-containing DNA fragment was glycosylated by using T4 phage β-glucosyltransferase (New England Biolabs) to generate the g5hmC-containg DNA fragment (D). For examining the cleavage activity of HHPV4I, different reaction buffers were used, as shown below. H: the HHPV4I reaction buffer (50 mM Tris-HCl, 100 mM NaCl, 5 mM $MnCl_2$, pH 7.9); 1.1: NEBuffer 1.1 (10 mM bis-Tris-propane-HCl, 10 mM $MgCl_2$, 0.1 mg/ml BSA, pH 7.0) supplemented with 5 mM $MnCl_2$; 2.1: NEBuffer 2.1 (10 mM Tris-HCl, 50 mM NaCl, 10 mM $MgCl_2$, 0.1 mg/ml BSA, pH 7.9) supplemented with 5 mM $MnCl_2$; NEBuffer 3.1 (50 mM Tris-HCl, 100 mM NaCl, 10 mM $MgCl_2$, 0.1 mg/ml BSA, pH 7.9) supplemented with 5 mM $MnCl_2$; CS: NEB CutSmart buffer (20 mM Tris-Ac, 50 mM KAc, 10 mM $Mg(Ac)_2$, 0.1 mg/ml BSA, pH 7.9) supplemented with 5 mM $MnCl_2$. The reactions (10 μl) contained 100 ng/μl of the indicated DNA substrate and 500 nM of HHPV4I and were incubated at 37°C for 1 h. MspJI was used as a control.

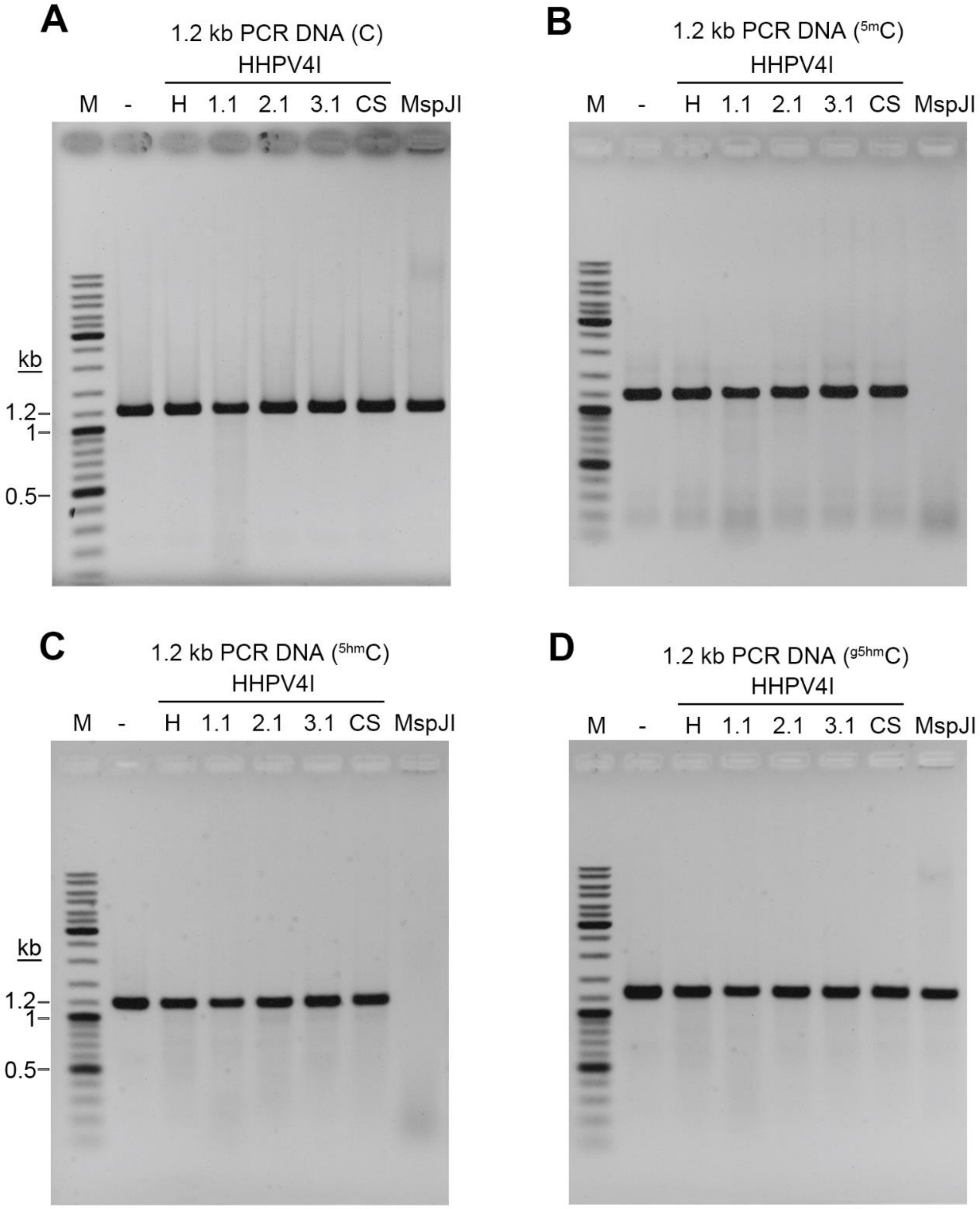
A
1.2 kb PCR DNA (C)
HHPV4I
M - H 1.1 2.1 3.1 CS MspJI
kb
1.2
1
0.5
B
1.2 kb PCR DNA (5mC)
HHPV4I
M - H 1.1 2.1 3.1 CS MspJI
C
1.2 kb PCR DNA (5hmC)
HHPV4I
M - H 1.1 2.1 3.1 CS MspJI
kb
1.2
1
0.5
D
1.2 kb PCR DNA (g5hmC)
HHPV4I
M - H 1.1 2.1 3.1 CS MspJI
